# Geological, geomorphological, and environmental insights into the Neoproterozoic Aswan granites, Egypt: remote sensing and radiological assessment

**DOI:** 10.1038/s41598-026-41770-2

**Published:** 2026-03-10

**Authors:** Gaafar A. El Bahariya, Ibrahim A. Salem, Gehad M. Saleh, Eman M. Ibrahim, Sameh E. Mohamed, Amal El Sarrag, Ali Shebl

**Affiliations:** 1 Geology Department, Faculty of Science, Tanta University, Tanta, 31527 Egypt; 2 Nuclear Materials Authority, Maadi, Cairo, Egypt; 3 Mineralogy and Geology Department, Debrecen University, Debrecen, 4032 Hungary

**Keywords:** Neoproterozoic Aswan granites, Egypt, Geology, Geomorphology, Remote sensing, Radioactivity, Quarrying, Landscapes, Cultural heritage, Unfinished Obelisk, Environmental sciences, Solid Earth sciences

## Abstract

The Neoproterozoic Aswan granites constitute a major post-collisional intrusive complex within the Egyptian Nubian Shield and represent one of Egypt’s most significant geological, geomorphological, and cultural landscapes. Integrated remote-sensing and radiological data are used to characterize their geological, geomorphological, and environmental attributes. Four granite suites are recognized in the Aswan area: greyish-black tonalites–granodiorites, coarse pink monzogranites–syenogranites, medium- to coarse-grained High Dam granites, and fine-grained granites. These lithologies exert a strong control on regional geomorphology, with structural fabrics—dominated by N–S and NE–SW joint sets and locally developed NE-trending shear zones—governing drainage patterns, landscape evolution, quarrying potential, and the distribution of radioelements. Integrated geomorphological and remote sensing analyses demonstrate that lithology and structural framework exert primary control on the Nile’s course and on the development of characteristic granite landforms, including inselbergs, exfoliation domes, joint-controlled valleys, steep canyons, and granitic island chains. PRISMA hyperspectral data further enhanced this framework by differentiating the rock units, delineating shear zones, and clearly identifying quarrying scars and associated landscape degradation. All remote-sensing interpretations were validated through detailed geological field investigations, ensuring accurate characterization of the granitic rocks and their modification by human activities. The granites show progressive differentiation from greyish-black granodiorites to coarse pink and fine-grained types, with U–Th–K enrichment and mean activities of ^238^U (77.23 Bq/kg), ^226^Ra (41.93 Bq/kg), ^232^Th (73.07 Bq/kg), and ^40^K (1281.3 Bq/kg), exceeding global averages. Radiological parameters record mean values of 131.35 nGy/h (absorbed dose), 0.16 mSv/y (annual effective dose), 275.82 Bq/kg (radium equivalent), hazard indices of 0.74 and 0.95, and a gamma index of 2.07. Fine-grained granites locally exceed indoor-use limits, High-Dam granites show moderate Th–K enrichment due to deformation, whereas greyish-black and most coarse pink granites (including Fila) remain within safety limits and are most suitable for quarrying. Overall, lithology and structural architecture—rather than external environmental factors—control mechanical behavior, landscape evolution, quarrying suitability, and radiological distribution across the Aswan granite province. Despite generally safe radiological levels, unregulated quarrying and rapid urban expansion have altered terrain and disrupted geomorphic integrity. These findings provide a framework for sustainable resource management, radiological safety evaluation, geomorphology-informed land-use planning, and preservation of Aswan’s unique geological and cultural heritage.

## Introduction

**Fig. 1:**
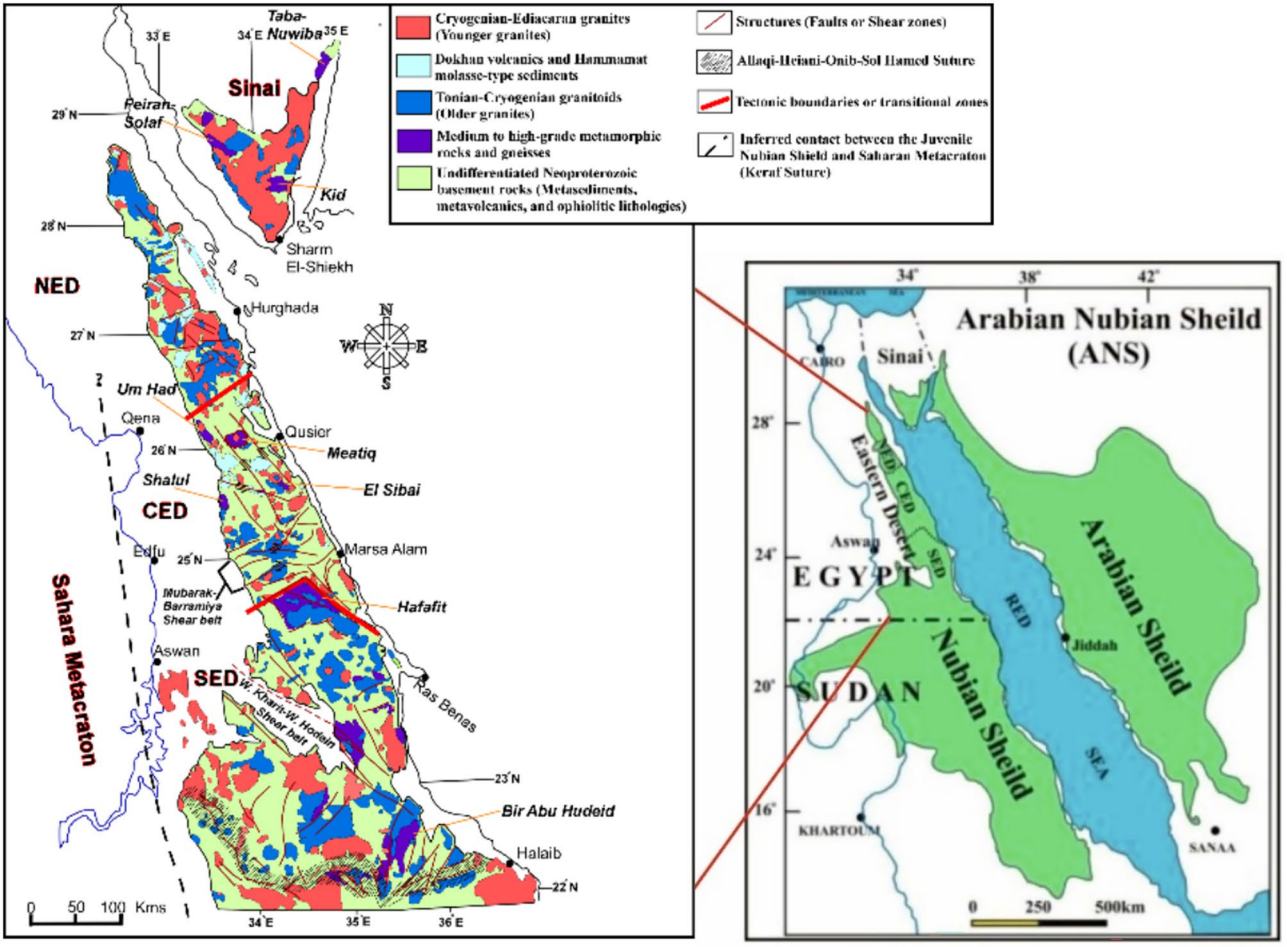
(**a**) The Arabian Nubian Shield (ANS)^7^; (**b**) Distribution of granitic rocks in the Egyptian Nubian Shield^8^.

Granitic rocks constitute the dominant component of the upper continental crust, accounting for nearly 87 vol%^1^, while the crust as a whole has an average geochemical composition comparable to granodiorite^2^. These rocks play a crucial role in defining the composition, evolution, and geodynamics of the Earth’s crust^3^. In Egypt, Precambrian basement rocks form the northeastern part of the Nubian Shield and are mainly exposed in the Eastern Desert and southern Sinai, where granitic plutons and batholiths are widespread^4^ (Fig. 1). Granites occupy approximately 57% of the total area of the Precambrian belt and are broadly classified into older grey granites (750–610 Ma) and younger pink granites (620–540 Ma)^5,6^. The older granites are mainly tonalitic and granodioritic, whereas the younger granites comprise monzogranites, syenogranites, and alkali feldspar granites.

The Neoproterozoic Aswan granites of southern Egypt form one of the most prominent geological provinces within the Arabian–Nubian Shield (ANS), marking a critical phase in the post-collisional evolution of the Pan-African orogeny. These granitic bodies are fundamental for reconstructing the tectono-magmatic history of northeastern Africa and stand as an enduring economic and cultural resource—the geological foundation of Egypt’s monumental architecture. These granites also provide an invaluable record of crustal stabilization, magmatic differentiation, and post-orogenic landscape development in the northern ANS. Their mineralogical diversity, structural fabrics, and geomorphic expression make the area an ideal natural laboratory for studying lithological–tectonic–geomorphological interactions within an arid shield terrain. Although classified as younger granites (YG) with ages ranging between 565 and 592 Ma^9^, other geochronological studies reported ages of 606 ± 2 Ma for the Aswan Tonalite and Monumental Granite and 595 ± 11 Ma for the High-Dam Granite^10^, suggesting multiple intrusive phases during late Pan-African crustal evolution.

Aswan granites were among the most prized stones in ancient Egyptian monumental architecture, valued for their strength and distinctive colors, and served as Egypt’s primary quarrying source for obelisks, statues, sarcophagi, and temples^11,12^. Petrographic and mineralogical correlations confirm that most New Kingdom plutonic building stones—especially coarse greyish-black and pink to red granites—were quarried directly from the Aswan granite field^13^. Today, however, intensive quarrying, rapid urban expansion, and infrastructure development have substantially modified the natural geomorphology, accelerating weathering and landscape degradation. This coexistence of outstanding geological heritage and increasing anthropogenic pressure makes Aswan a critical setting for evaluating human impacts on granitic terrains.

Granitic rocks contain naturally occurring terrestrial radionuclides, primarily ^238^U, ^232^Th, and ^40^K, with average concentrations of ~ 5 ppm for U and 15 ppm for Th^14^. The decay of these elements generates natural gamma radiation, which can pose environmental or occupational hazards if locally concentrated (Verdoya et al. 2009). Assessing their activity concentrations is essential for radiological safety, particularly in quarrying and urbanized areas. In Egypt’s Eastern Desert, younger granites, including those of Aswan, are typically more uranium-rich than other rock types^15^. Nevertheless, key knowledge gaps persist: the geological heterogeneity of Aswan granitoids is poorly constrained due to limited field studies and geochemical data, leaving uncertainties in magmatic evolution and granite classification. Likewise, the geomorphological and structural controls on landscape development and Nile drainage—particularly the influence of Aswan granites—remain insufficiently understood. Systematic environmental and radiological assessments are scarce, and anthropogenic pressures from rapid urbanization, infrastructure expansion, and unregulated quarrying have accelerated weathering, altered surface runoff, and progressively degraded the landscape.

Recent advances in remote sensing have enhanced geological and environmental investigations, particularly in complex and inaccessible terrains^16–18^. The integration of hyperspectral data, such as that from PRISMA, with analytical techniques including False Color Composites (FCC), SMACC endmember extraction, and curvature analysis, enables precise lithological discrimination, identification of geomorphological features, and monitoring of anthropogenic impacts^19^. When combined with field and petrographic observations, these methods provide a powerful geospatial framework for interpreting lithological variation, erosion dynamics, and environmental changes in regions such as Aswan.

This study employs an integrated multidisciplinary framework combining geological field investigations, petrographic analysis, geomorphological characterization, hyperspectral and multispectral remote sensing, and radiological assessment to investigate the Neoproterozoic Aswan granites, Egypt. By integrating ground observations with PRISMA hyperspectral imagery, SMACC endmember extraction, and curvature-based terrain modeling, the study achieves high-resolution discrimination of granite lithologies, delineates structural controls, and clarifies geomorphological evolution and its influence on drainage development and weathering processes, while documenting the spatial impacts of quarrying and urban expansion. Radiological measurements of ^238^U, ^232^Th, ^226^Ra, and ^40^K provide critical insight into radioelement distribution and associated radiological hazards across different granite types. Collectively, these datasets enable characterization of lithological and structural variability, assessment of geomorphic controls on landscape development, evaluation of radiological risks relevant to human activity and quarrying, and the establishment of a scientifically grounded framework for sustainable resource management, land-use planning, and environmental protection in the Aswan region.

By synthesizing geological field observations, petrographic analyses, hyperspectral remote sensing, and radiometric measurements, this work provides an integrated understanding of the interplay between lithology, geomorphological processes, and human activities in one of Egypt’s most geologically and culturally significant regions. The outcomes support sustainable utilization of granite resources, risk-informed quarrying and land-use planning, and the long-term preservation of the Aswan geoheritage landscape.

## Study area and geological setting

The Aswan region of southern Egypt was selected for this study because it represents one of the most geologically, environmentally, and culturally significant areas of the Nubian Shield. Its well-exposed Neoproterozoic Aswan granites provide an exceptional setting for examining post-collisional magmatism, landscape evolution, and the structural controls exerted on the Nile River and surrounding arid-zone geomorphology. Environmentally, these granitic terrains contain naturally elevated U, Th, and K concentrations, making Aswan a key site for assessing natural radioactivity amid active quarrying, rapid urban growth, and extensive granite use in construction. Culturally, Aswan has supplied monumental granite for temples, obelisks, and statues for millennia, and modern quarrying continues to shape both the economy and the surrounding UNESCO-listed heritage landscape. With modern remote sensing enabling high-precision mapping, Aswan stands out as a scientifically valuable and culturally sensitive area ideal for integrated geological, geomorphological, and environmental investigation.

**Fig. 2:**
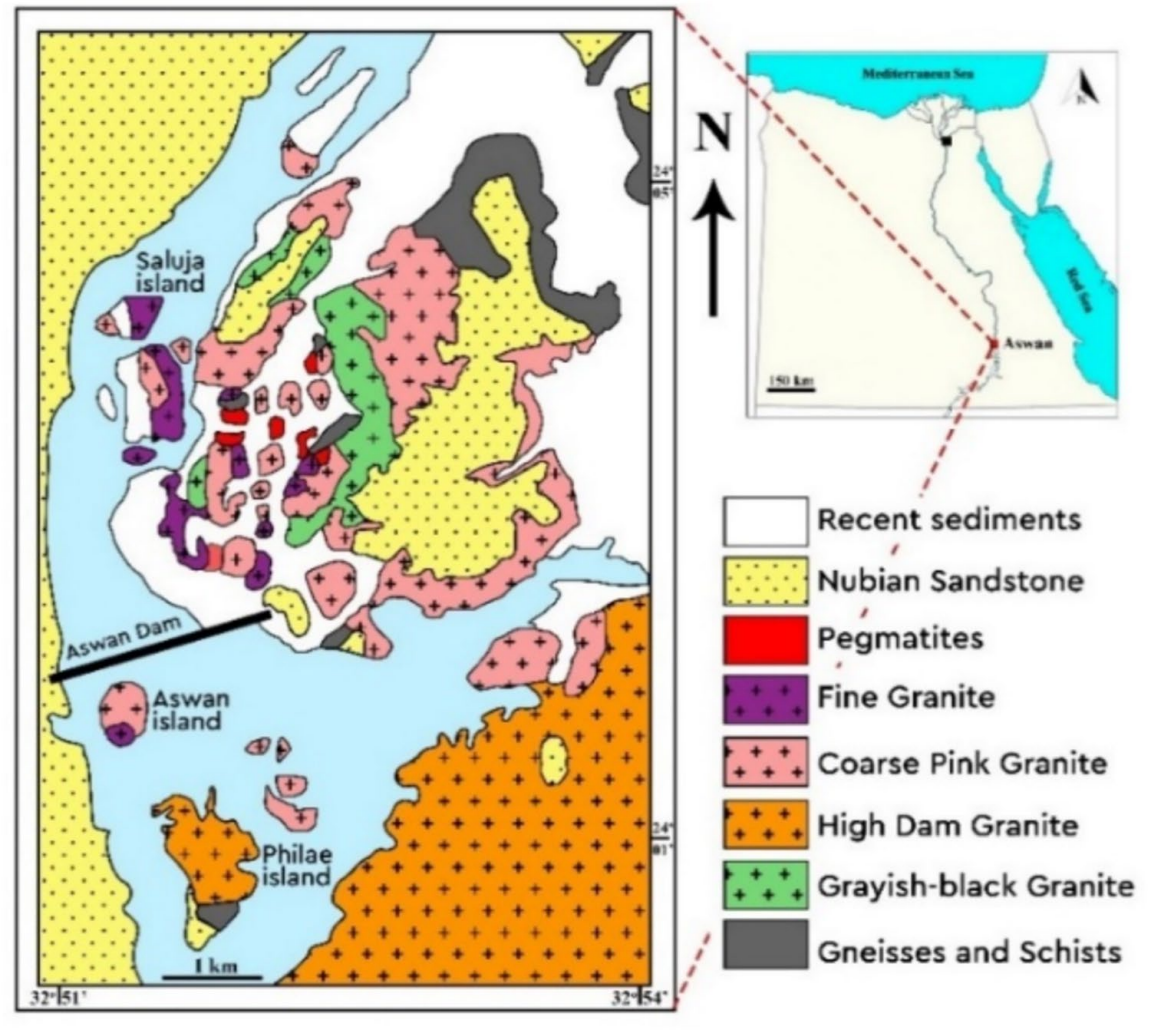
Geological map of Aswan area^20^. Inset showing the location map of Aswan area on the map of Egypt.

Geographically, the study area lies in the western sector of the Southern Eastern Desert between latitudes 24° 05′–18° 05′ N and longitudes 32° 53′–26° 07′ E (Fig. 2), along the northwestern margin of the Arabian–Nubian Shield (ANS), the northernmost segment of the Pan-African orogenic belt. It contains extensive exposures of Precambrian and Phanerozoic rocks, dominated by Neoproterozoic granitic bodies that define the structural and geomorphic framework. These granites strongly influence the Nile’s course, creating channel constrictions and island clusters where the river encounters resistant bedrock. The region comprises multiple granite varieties forming elongate north–south-trending bodies from Aswan to El Shellal, ranging from coarse-grained grey tonalites and granodiorites to coarse-grained pink monzogranites, syenogranites, and fine-grained granites. Coarse pink granites are the most widespread and form the dominant geomorphological features. The granitic masses intrude older schists and gneisses along sharp contacts and, in places south and west of Aswan, are unconformably overlain by Phanerozoic Nubia Sandstone formations.

The Aswan region is characterized by a rugged terrain of low hills and moderate-relief mountains, with granitic hills and domes forming the structural foundation of the city and its surroundings. The Nile River cuts directly through the crystalline basement, creating granite islands such as Sehel, Saluja, and Philae—distinctive examples of river incision into resistant granitic rocks and structurally guided channel alignment. The landscape is dissected by major NE–SW, NW–SE, and E–W fault systems that bound the granitic massifs and strongly influence drainage evolution. Joint sets, exfoliation surfaces, and fault zones further shape slope morphology, runoff patterns, and weathering intensity, producing geomorphic features including exfoliation domes, tors, and isolated inselbergs on broad pediplains. Climatically, the area lies within the hyper-arid belt of North Africa, receiving less than 2 mm of annual rainfall. Intense solar radiation, strong diurnal temperature fluctuations, and prolonged dryness promote mechanical weathering and limit chemical alteration, leaving granitic surfaces exceptionally well exposed—ideal for geological, geomorphological, and remote sensing investigations.

## Materials and methods

This study applies an integrated, state-of-the-art methodological framework to address its multidisciplinary objectives. Conventional geological mapping and petrographic analysis establish the basis for rock units identification and structural interpretation, while remote sensing techniques using PRISMA imagery improve lithological discrimination, geomorphological mapping, and environmental evaluation. Geomorphological analysis examines weathering patterns, erosional features, and landform development in relation to structural controls and human influence. Complementary radiological investigations characterize the environmental and radiometric properties of the Aswan granites. Together, these approaches provide a coherent methodology that links geological, geomorphological, and radiometric datasets within a unified analytical system.

### Field work and sampling

Fieldwork was carried out during two field trips by Professor Gaafar El Bahariya, during which field observations and measurements of structural elements were made. More than one hundred samples were collected from different types of Aswan granites at various sites in the Aswan area, including some granite quarries in Aswan city. Over 500 photos of field observations, geomorphological features, and landscapes were taken by Professor El Bahariya, the first author of this scientific paper and the principal supervisor of Amel El Sarrag’s Master’s thesis, of which this paper is a part.

Field investigations and systematic sampling of all active and abandoned Aswan quarries targeted major granite types based on geological characteristics, quality, and environmental relevance. Surveyed sites included the historic coarse-grained pink granite quarry at the Unfinished Obelisk, two greyish-black granite quarries at Ibrahim Basha, and both active and inactive coarse pink granite quarries, notably the active Chinese and El Mesalla sites and an inactive quarry near El Mesalla. Numerous samples and field photographs, taken by Professor El Bahariya, supported the geological assessment.

### Thin sections

Thirty thin sections representing the different granite types from various Aswan sites and quarries were prepared and examined under transmitted polarized light microscopy. This analysis focused on classifying the granites, characterizing their mineralogical composition, and documenting their key petrographic features through detailed photomicrographs.

### Remote sensing

PRISMA hyperspectral data (234 VNIR and SWIR bands) enabled detailed lithological discrimination, and environmental monitoring, detecting subtle compositional variations beyond conventional multispectral sensors. Combined with high-resolution ESRI LULC maps from Sentinel-2 (2017 and 2023), they classified major land-cover types—urban, vegetation, water, and barren land—and assessed human impacts on Aswan’s geological and geomorphological settings.

False Color Composite (FCC) imagery emphasized lithological contrasts, vegetation, and urban patterns, while the SMACC algorithm extracted key spectral endmembers to identify dominant surface materials. Topographic analyses evaluated surface roughness and structural variability, delineating geological boundaries, faults, and erosion-prone zones, supporting an integrated geological and environmental interpretation of the Aswan area.

### Radiometric methods

Thirty-six granite samples were collected from three Aswan occurrences**:** greyish-black granites, coarse pink granites, and fine granites from Aswan city and quarry sites (27 samples)**;** coarse pink granites from the Fila area (5 samples); and medium to coarse granites from the High Dam area (4 samples)**.** All samples were crushed, sieved to –60 mesh, sealed in 200 cm^3^ polyethylene beakers, dried at 105 °C for 24 h, and analyzed using a NaI(Tl) gamma-ray spectrometer following standard energy and sensitivity calibration. Radionuclides were quantified using three energy regions of interest (ROIs) corresponding to ^234^Th, ^214^Pb, ^212^Pb, and ^40^K, representing U, Ra, Th, and K, respectively. ^238^U was determined from the 92.6 keV gamma line of ^234^Th, while ^226^Ra and ^232^Th were measured using the 352 keV (^214^Pb) and 238.6 keV (^212^Pb) transitions. ^40^K was measured at 1460.8 keV, with analytical uncertainties of 10–15%.

### Radiological Hazard Indices

The absorbed gamma dose rate at 1 m above ground was calculated following^21^:

1 $${\mathrm{D}}\left( {{\mathrm{nGy}}/{\mathrm{h}}} \right) = 0.{\text{462 A}}_{{\mathrm{U}}} + 0.{6}0{\text{4 A}}_{{{\mathrm{Th}}}} + 0.0{\text{417 A}}_{{\mathrm{K}}}$$

where A_U_, A_Th_, and A_K_ are the activity concentrations of ^238^U, ^232^Th, and ^40^K** (**Bq kg^−1^), respectively.

The annual effective dose (mSv/y) was estimated using a 0.7 Sv/Gy dose conversion factor and an outdoor occupancy factor of 0.2:

$${\text{Effective dose }}\left( {{\mathrm{mSv}}/{\mathrm{y}}} \right) = {\text{Dose rate}}\left( {{\mathrm{nGy}}/{\mathrm{h}}} \right) \times {876}0{\text{ h}} \times 0.{2} \times 0.{7}\left( {{\mathrm{Sv}}/{\mathrm{Gy}}} \right) \times {1}0^{ - 6}$$

Radium equivalent activity (Raeq) was calculated to assess combined gamma dose from ^226^Ra, ^232^Th, and ^40^K:

$${\mathrm{Ra}}_{{{\mathrm{eq}}}} = {\mathrm{A}}_{{{\mathrm{Ra}}}} + \left( {{1}0/{7}} \right){\mathrm{A}}_{{{\mathrm{Th}}}} + \left( {{1}0/{13}0} \right){\mathrm{A}}_{{\mathrm{K}}} \quad \left( {{\text{limit of 37}}0{\text{ Bq}}/{\mathrm{kg}}} \right)$$

The internal (H_in_) and external (H_ex_) hazard indices evaluate radon-related and gamma exposure risks, respectively^22,23^:

$${\mathrm{H}}_{{{\mathrm{in}}}} = {\mathrm{A}}_{{\mathrm{U}}} /{185} + {\mathrm{A}}_{{{\mathrm{Th}}}} /{259} + {\mathrm{A}}_{{\mathrm{K}}} /{481}0 < {1}, \;{\mathrm{H}}_{{{\mathrm{ex}}}} = {\mathrm{A}}_{{{\mathrm{Ra}}}} /{37}0 + {\mathrm{A}}_{{{\mathrm{Th}}}} /{259} + {\mathrm{A}}_{{\mathrm{K}}} /{481}0 \le {1}$$

The gamma activity concentration index (Iγ) estimates gamma radiation risk^24^:

$${\mathrm{I}}{ gamma } = \left( {{1}/{15}0} \right){\mathrm{A}}_{{\mathrm{U}}} + \left( {{1}/{1}00} \right){\mathrm{A}}_{{{\mathrm{Th}}}} + \left( {{1}/{15}00} \right){\mathrm{A}}_{{\mathrm{K}}}$$

All activity concentrations refer to ^238^U, ^226^Ra, ^232^Th, and ^40^K (Bq/kg).

## Results

### Geological characteristics

The study area lies mainly on the eastern Nile bank, extending across Aswan town and southeastward where several granite types are exposed (Fig. 2). These granites form low hills and numerous islands—most notably the Saluja–Sehel and Philae islands—and intrude older schists, gneisses, amphibolites, and migmatites along sharp contacts, locally cut by pegmatite veins. Field relations show a clear intrusive sequence: greyish-black granites → coarse pink granites → fine-grained Saluja–Sehel granites, while the High-Dam granites occur as extensive bodies near the dam. Based on geological setting, field relations, and grain size, the Aswan granites are divided into four main types: (1) greyish-black granites, (2) coarse pink granites, (3) High-Dam granites, and (4) fine greyish-red granites.

**Fig. 3:**
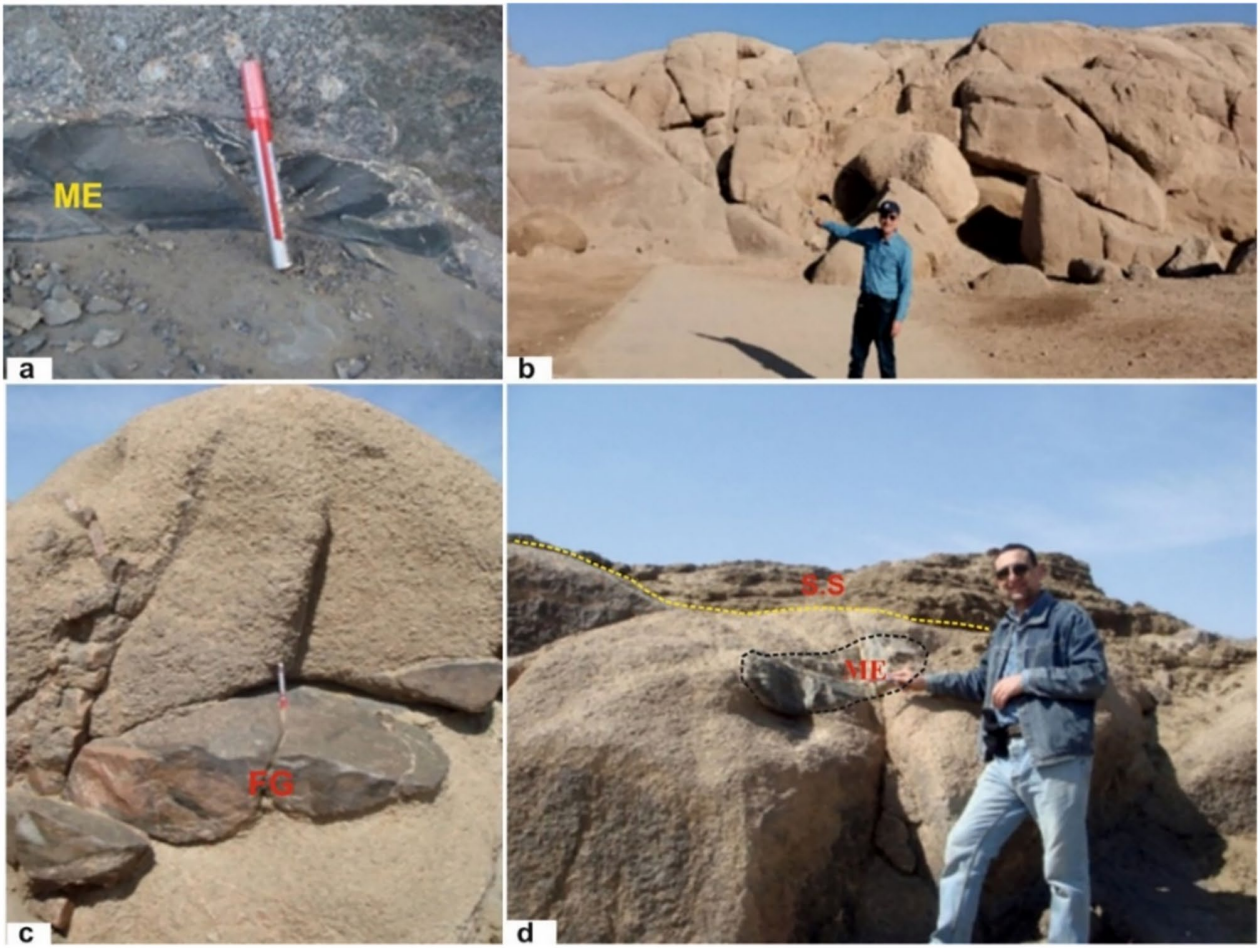
(**a**) Grayish black granites with pinkish white phenocrysts enclose elongate mafic enclaves (ME); (**b**) Coarse red granites of the Un finished obelisk site in aswan city. (**c**) Coarse pink granite intruded by dyke -like fine grained granite (FG) (looking E) in Suheil Ghazal island; (**d**) Coarse-grained pink granite enclose mafic enclaves (ME), which displaced along strike slip fault and unconformably overlined by phanerozoic sandstone (SS) (photo looking W). All photographs included in this manuscript feature Professor Gaafar El Bahariya, the first author of this study, who has given full informed consent for their publication.

Greyish-black granites form elongated N–S sheet-like bodies, especially in the Gabal Ibrahim Basha area, where they constitute major exposures. They include coarse-grained to porphyritic tonalites, granodiorites, and quartz monzonites with white K-feldspar phenocrysts and abundant quartz. Elongated dyke-like mafic enclaves and xenoliths are common, occurring as isolated or clustered bodies aligned with regional structures. Many appear along granite margins, showing sharp contacts and limited hybridization (Fig. 3a).

Coarse pink granites are widespread between Aswan and Shellal, forming several Nile islands—including Philae Island—and also occurring on the mainland at Gabal El Messala in central Aswan city (Fig. 3b). They are coarse to very coarse grained, enriched in pink K-feldspar, and commonly contain lens-shaped to ovoid mafic enclaves. On Saluja–Sehel Island, these granites are intruded by later fine-grained granitic and pegmatitic sheets and dykes, contain abundant mafic enclaves, and form low rounded hills partly overlain by Phanerozoic sandstones (Fig. 3c, d). A dominant N–S joint system controls their weathering and landform development. They are composed of monzogranites and syenogranites with large pink K-feldspar phenocrysts.

**Fig. 4:**
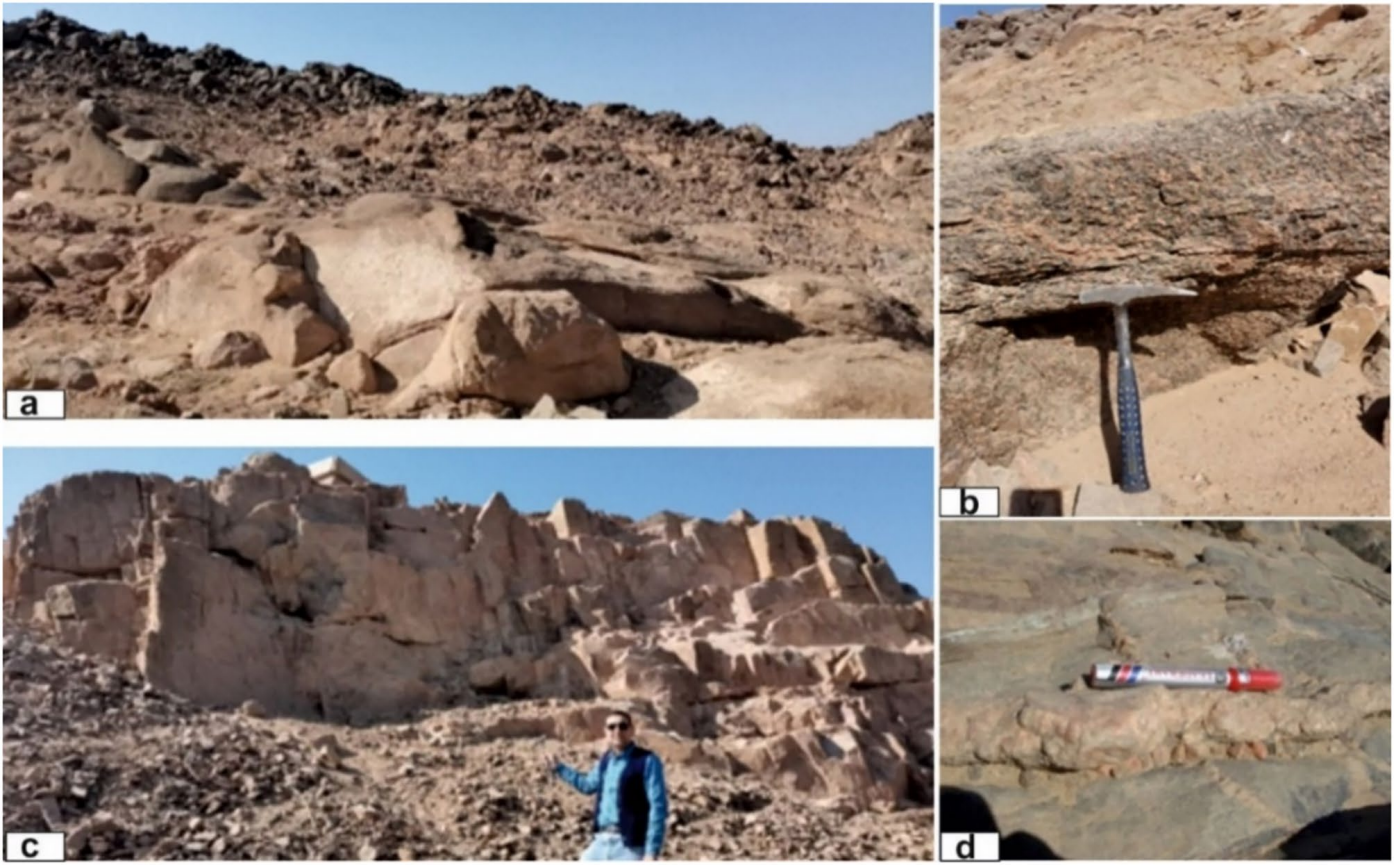
(**a**) High dam granites exhibiting dome-shaped granite hill sparsely covered by detached rounded and angular boulders (Looking W); (**b**) Sheared monzogranit (Mylonitic granites) along NE shear zone; (**c**) Jointed fine-grained granites on the eastern bank of the Nile river north Aswan dam (looking NE); (**d**) Pegmatitic viens and flow parallism of fine grined granites during intrusion into the coarse pink granite into the countru rocks, Saluga-jazal island.

High-Dam granites extend north and northeast of the High Dam as low to moderately elevated hills composed mainly of monzogranites and syenogranites, with local granodiorite patches. They vary in color from deep red to greyish red and display notable compositional heterogeneity. Many exposures exhibit spheroidal weathering and localized deformation related to a NE-trending shear zone, which produces foliation, shearing, and intense fracturing (Fig. 4a, b). Mafic-rich xenoliths and schistose enclaves—rounded, lens-shaped, or elongated—are abundant and commonly aligned parallel to the shear zone.

Fine greyish-red granites occur along the Nile flanks and on Saluja–Sehel Island as irregular bodies and narrow dyke-like intrusions that sharply cut the coarse pink granites. They exhibit well-developed joint and fracture sets, and several exposures show gneissose fabrics defined by the preferred alignment of biotite and other ferromagnesian minerals (Fig. 4c, d). These granites are consistently fine-grained, pink to greyish-pink in color, and share monzogranitic–syenogranitic compositions with the coarse pink granites, differing primarily in grain size and structural characteristics.

### Geomorphology and landforms

**Fig. 5:**
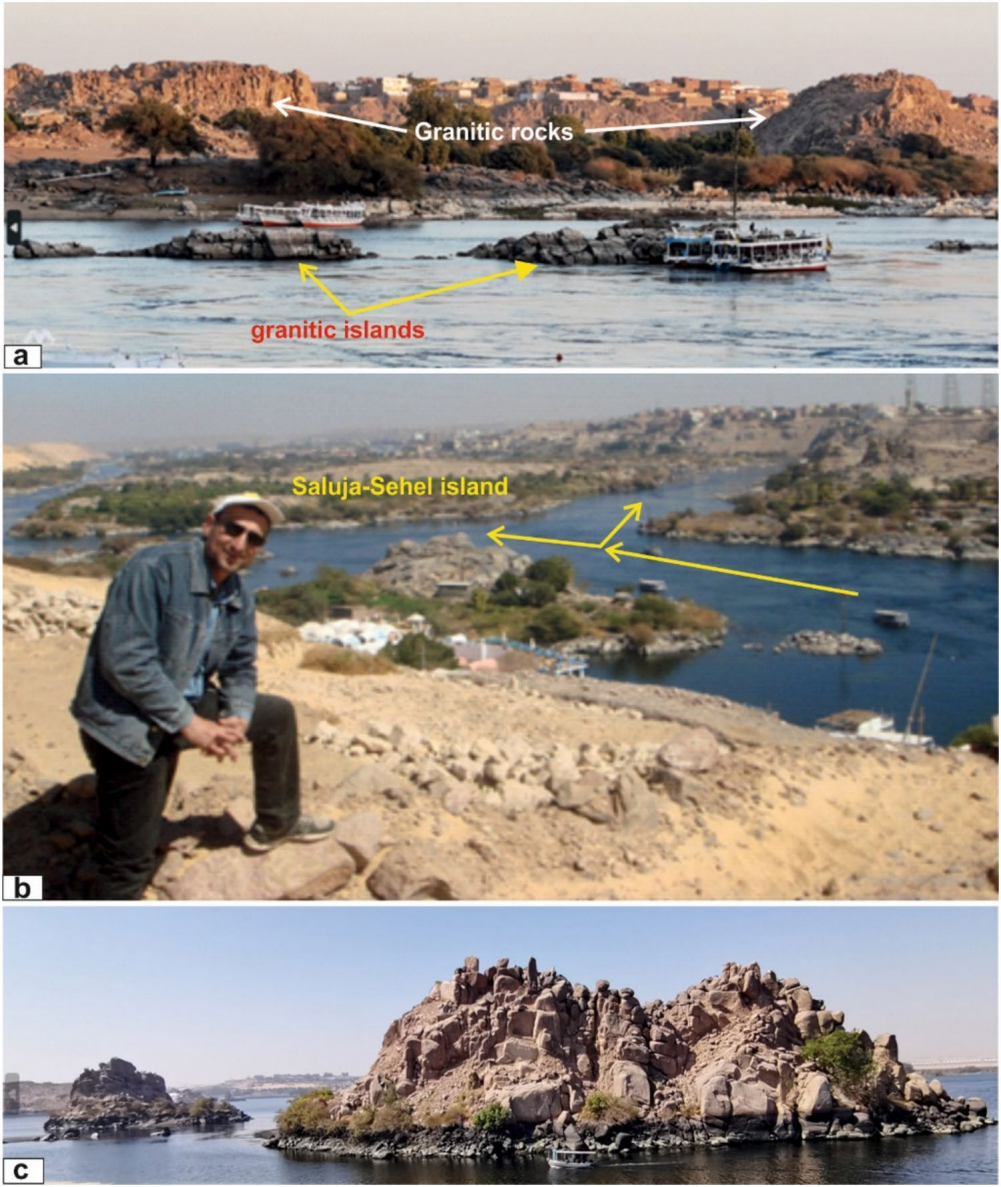
Geomorphology and landscapes of Aswan granites. (**a**) Granitic rocks on the eastern bank of the river and isolated small islands of weatherd granite (north Aswan dam with, Looking E); (**b**) General view shows some of the granitic islands, where the course of the Nile River is deviated around the granitic islands (looking NE); (**c**) Photo of Philae islands showing joints and blocks of granites (up) and weathered and altered granite surfaces (down) (Looking N).

The study area comprises low to moderately elevated granite hills forming the main bedrock beneath Aswan city and extending along the eastern bank of the Nile, together with numerous granite islands of varying size and elevation—most prominently Saluja–Sehel and Philae Islands. The geomorphology is primarily governed by the interaction between these exposed granitic masses and the Nile River. The high hardness and resistance of the granites force the river to divert around the outcrops or carve narrow gorges where these resistant units intersect the channel, resulting in cataracts, constricted reaches, and the development of multiple granite islands (Fig. 5a–c).

**Fig. 6:**
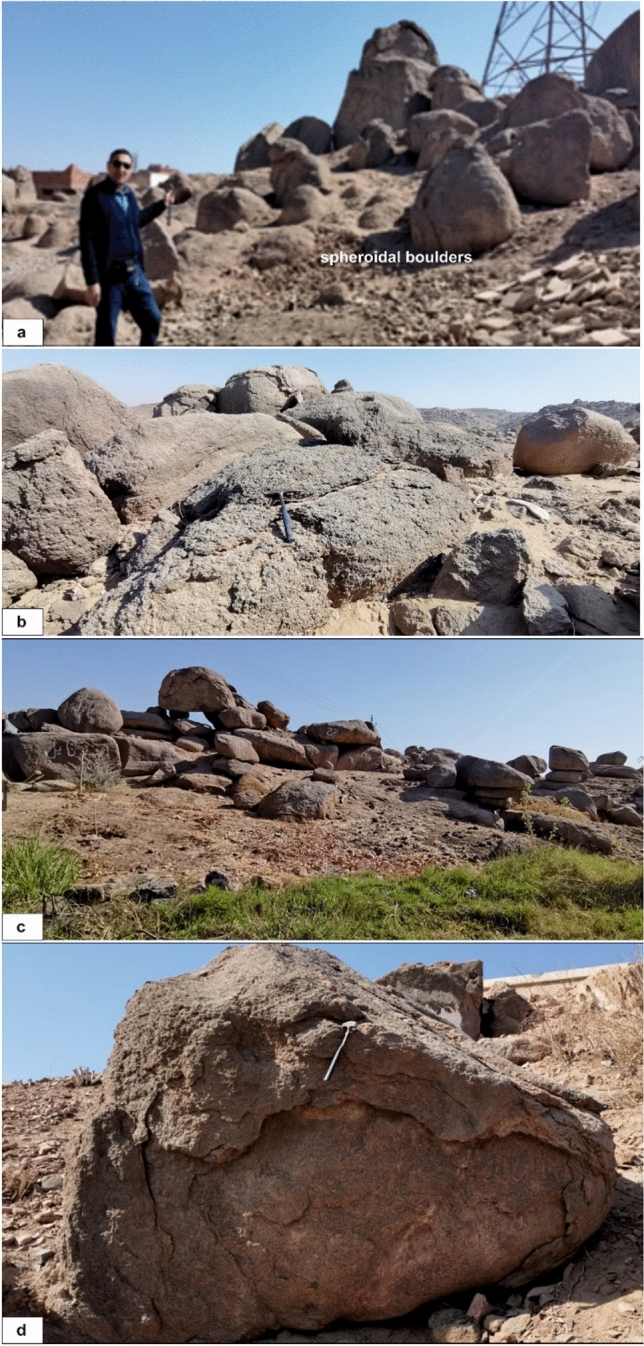
(**a**) Spheroidal and exfoliation weathering in coarse pink granites between residential buildings in Aswan City (Looking NE); (**b**) Exfoliation, domal, and spheroidal structures in coarse pink granites east of the Philae Islands on the Nile’s eastern bank (Looking SE); (**c**) joint-controlled spheroidal and dislocated blocks of coarse red granites, (Looking SW); (**d**) Exfoliation of red carse granites in Aswan city area.

The Aswan coarse pink granites show moderate to high degrees of physical weathering and develop a range of joint-controlled landforms, including variably sized boulders, spheroidal weathering, woolsack structures, and exfoliation and domal structures (Fig. 6a–d). While some boulders can exceed 10 m in length, most typically range from 1 to 2 m.

**Fig. 7:**
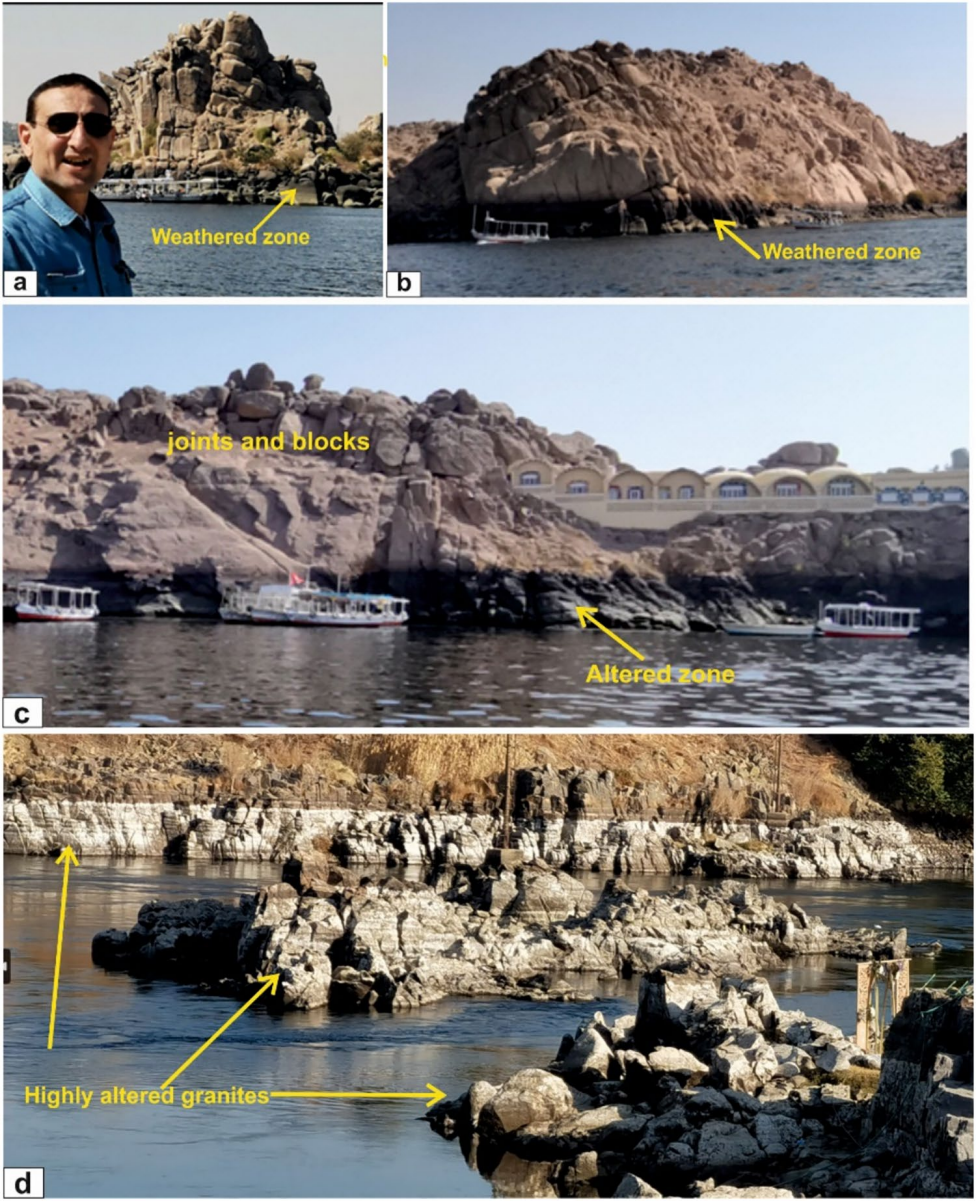
Jointed and weathered landforms of Aswan granites. (**a**) Coarse pink granites at Philae Island with N–S joints and rounded or dislocated blocks above and chemically weathered surfaces below. (Looking N); (**b**) Coarse pink granites at Philae Island showing lower weathered marker zones and upper jointed blocks (Looking SE); (**c**) Granitic rocks on the eastern bank of the river as a base foundation of modern buildings showing joint-controlled spheroidal and dislocated blocks (up) and chemically weathered zone (down) north Aswan dam (Looking E); (**d**) Granitic islands strongly weathered and altered by the continuous flow of Nile River water (view looking SE).

A dominant N–S joint system dissects the granites, producing large detached blocks that are especially prominent along upper slopes and hill crests (Fig. 7a). In contrast, granite exposures along the Nile—particularly on islands and riverbanks—are more affected by chemical weathering due to continuous interaction with river water. Periodic annual floods intensify surface alteration, discoloration, and the formation of well-defined weathering fronts, which over successive flood cycles have progressively deepened and expanded the altered zones along the granite outcrops (Fig. 7b, c, d).

Moreover, many of these granite outcrops now form the natural foundation for modern construction across Aswan, where buildings are often built directly on the exposed bedrock. Remnant granite hills continue to rise above the urban landscape, providing prominent geomorphic and visual features within the city.

### Aswan granite quarry sites

The Aswan region contains numerous ancient and modern granite quarries, reflecting its long-standing role as a major source of building stone in Egypt. Extensive fieldwork across multiple quarry locations documented representative granite samples, field relationships, quarrying traces, and environmental conditions. Many granite bodies exhibit spheroidal boulders, naturally shaped by joints, which were exploited as ancient quarrying targets. Dark grey granodiorite quarries were particularly significant, used from the Predynastic through Roman periods for statues, stelae, architectural elements, and monumental structures (Nicholson and Shaw 2000). The surrounding landscape preserves ancient extraction pits, unfinished blocks, and tool marks, now modified by modern urbanization, erosion, and ongoing quarrying.

**Fig. 8:**
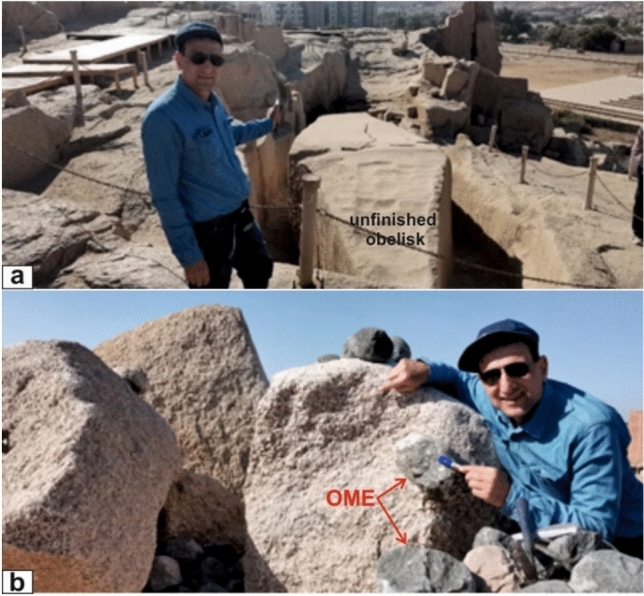
(**a**) Unfinished Obelisk, Aswan City quarry: ~ 42 m long, oriented N20° E and dipping ~ 30° SW—geometry first recorded by Professor El Bahariya in this study (view looking SW); (**b**) Ovoidal mafic enclaves hosted in coarse-grained pink granites at the Unfinished Obelisk quarry in Aswan City.

This study provides new observations, recorded for the first time by Professor El Bahariya during his fieldwork, of the ancient quarry containing the unfinished obelisk, carved from coarse pink granite. The obelisk measures ~ 42 m in length, strikes N20° E, and dips ~ 30° SW, remaining attached to the parent rock (Fig. 8a). The surrounding coarse-grained pink monzogranite contains conspicuous ovoid mafic enclaves, both isolated and clustered, which, along with natural joint sets and variable weathering, influenced site selection and the feasibility of extracting large monolithic blocks (Fig. 8b).

**Fig. 9:**
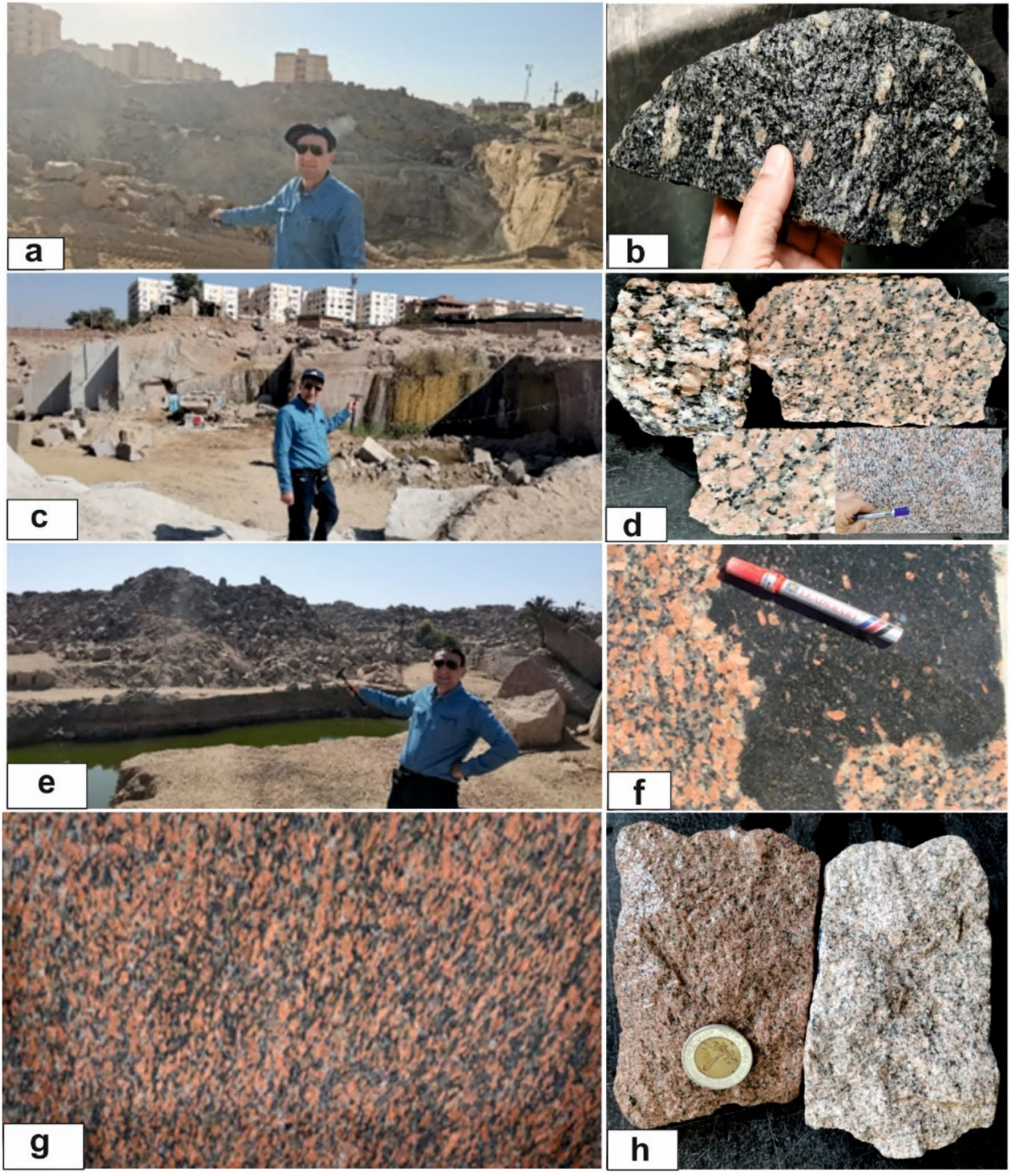
Active and inactive quarry sites and representative hand specimens of Aswan granites. (**a**) Greyish-black granite quarry at the Ibrahim Basha site; (**b**) Polished slabs Greyish-black granite with large K-feldspar and quartz phenocrysts; (**c**) Active coarse pink granite quarry near the El Mesalla Obelisk; (**d**) Hand specimens of various red and pink, coarse-grained Aswan granites; (**e**) Inactive coarse pink granite quarry near El Mesalla Hospital, with a stagnant water pool caused by improper recent quarrying; (**f**) Mafic enclave hosted within red coarse granite, displaying sharp, irregular contacts in a recent active quarry; (**g**) Polished slab of North High-Dam deformed granite; (**h**) Hand specimens of fine-grained granites.

Most samples were collected from principal quarries extracting coarse pink granite—the dominant building stone historically and in modern Aswan—with additional samples from dark tonalitic–granodioritic quarries and fine-grained late-stage intrusions cutting the coarse pink granite (Fig. 9a–d). This dataset represents the full range of granite types, supporting assessment of lithological variability, weathering, and environmental and radiological implications of quarrying.

**Grayish-black granite** is coarse- to medium-grained and primarily extracted from urban quarries, notably Ibrahim Basha (Fig. 9a). Its dark color reflects abundant mafic minerals (biotite and hornblende), with speckled white–pink K-feldspar and quartz (Fig. 9b). Well-developed N–S and NE–SW joints control block geometry, facilitate extraction, and expose fresh surfaces revealing internal fabric, mineralogical variations, and structural features. Its high strength and massive structure make it widely used in architecture and construction.

**Aswan’s coarse pink granite** is the region’s most important and extensively quarried building stone, forming the foundation of a long-standing quarrying tradition. The main quarries, located in the low mountains of central Aswan—particularly near the Unfinished Obelisk—show extraction from antiquity to the present and include both active industrial sites, such as a large Chinese-operated quarry with advanced cutting technologies, and abandoned workings preserving ancient tool marks, trenches, and unfinished blocks (Fig. 9c, e). Prominent N–S joint systems control quarry geometry and block size, while weathering along these joints in recently abandoned sites produces exfoliation surfaces, rounded boulders, and small water-filled depressions that gradually reshape the landscape. The granite is very coarse-grained, with pink to reddish tones dominated by large K-feldspar phenocrysts intergrown with quartz, plagioclase, and biotite, and exhibits red, black, and orange color variations along with common subrounded to irregular mafic enclaves (Fig. 9d, f), features that contribute to its visual appeal and enduring architectural significance.

**High Dam granites** include coarse- to medium-grained greyish-red to red monzogranites and granodiorites, some locally deformed with gneissic fabrics and distinctive color banding, enhancing their aesthetic appeal for ornamental and decorative uses (Fig. 9g). Fine-grained granites, mainly along the eastern Nile bank, expose greyish-pink monzogranite with a uniform, closely intergrown texture of quartz, K-feldspar, plagioclase, and minor biotite and muscovite, clearly distinct from coarser units and producing their characteristic greyish-pink coloration (Fig. 9h).

### Petrographic features

**Fig. 10:**
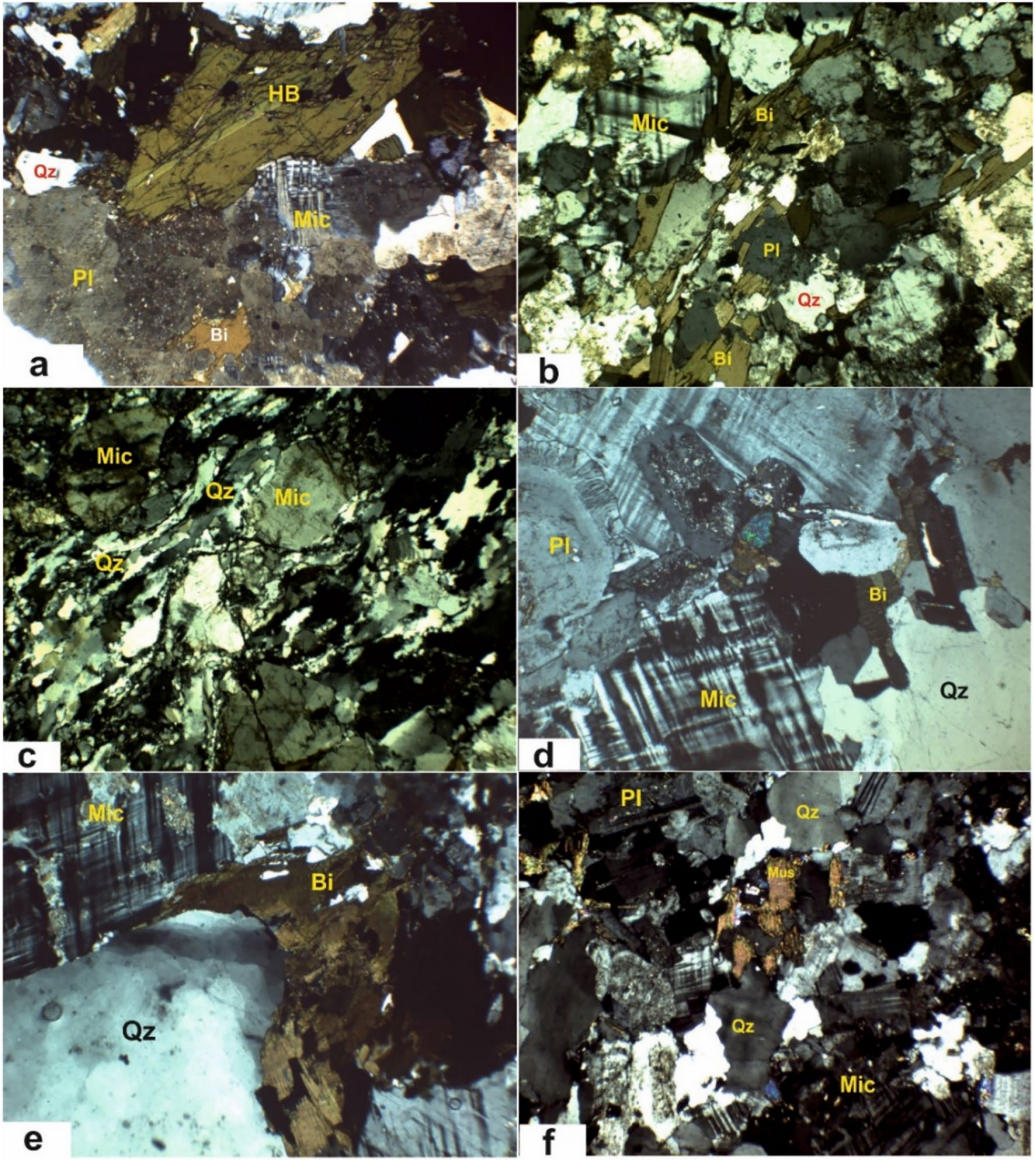
Photomicrographs of the studied Aswan granites: (**a**) granodiorites; (**b**) gneissose granodiorites; (**c**) sheared syenogranites; (**d**) coarse-grained monzogranites; (**e**) coarse-grained syenogranites; and (**f**) fine-grained monzogranites (crossed Nicols, CN, 4 ×). Mineral abbreviations: Qz = quartz; Pl = plagioclase; Mic = microcline; Bi = biotite; Hb = hornblende; Mu = muscovite.

Petrographic analysis was conducted to classify Aswan granite types and document their mineralogical and textural characteristics:

**Grayish-black granites** are massive, coarse-grained, and grey to dark grey, speckled with quartz and feldspar (Fig. 10a). They consist mainly of quartz, plagioclase, K-feldspar, hornblende, and biotite, with secondary minerals including chlorite, sericite, Fe-oxides, calcite, and kaolinite; apatite, zircon, and sphene occur as accessories. Textures are hypidiomorphic with perthitic and anti-perthitic features.

**High Dam gneissose granodiorites** are medium- to coarse-grained, reddish-grey to red, mostly massive but locally foliated (Fig. 10b). Composed of plagioclase, quartz, K-feldspar, and abundant biotite, they contain accessory zircon, sphene, and apatite, with secondary sericite, kaolinite, and chlorite. Biotite flakes form bands, producing gneissose textures; large plagioclase and K-feldspar phenocrysts impart a porphyritic appearance.

**Mylonitic granites** exhibit pronounced cataclasis while partially preserving original granitic textures (Fig. 10c). Composed of quartz, K-feldspar, plagioclase, and subordinate biotite, with secondary sericite and chlorite, and accessory Fe-oxides, zircon, and apatite. Mylonitic textures feature stretched quartz aggregates and biotite flakes wrapping feldspars.

**Pink coarse granites** (Aswan city and Fila occurrence) include monzogranites and syenogranites, composed mainly of quartz, K-feldspar, plagioclase, biotite, and subordinate hornblende (Fig. 10d–e). Secondary minerals are chlorite, sericite, kaolinite, Fe-oxides, and sphene; zircon and apatite are accessory. Textures are hypidiomorphic, perthitic, antiperthitic, myrmekitic, and micrographic; large pink K-feldspar grains impart a porphyritic texture. Slight shearing and cataclasis occur but granitic textures are largely preserved.

**Fine-grained monzogranites** are massive, reddish-grey, composed of quartz, K-feldspar (microcline and perthite), plagioclase, with subordinate biotite and muscovite (Fig. 10f). Secondary minerals include chlorite, sericite, Fe-oxides, kaolinite, epidote, and carbonate; zircon and apatite are accessory. They exhibit hypidiomorphic textures, with occasional biotite bands producing a gneissose appearance, and intergrowths of K-feldspar and plagioclase forming perthitic, antiperthitic, and myrmekitic textures.

### Remote sensing findings

#### Geological analysis

**Fig. 11:**
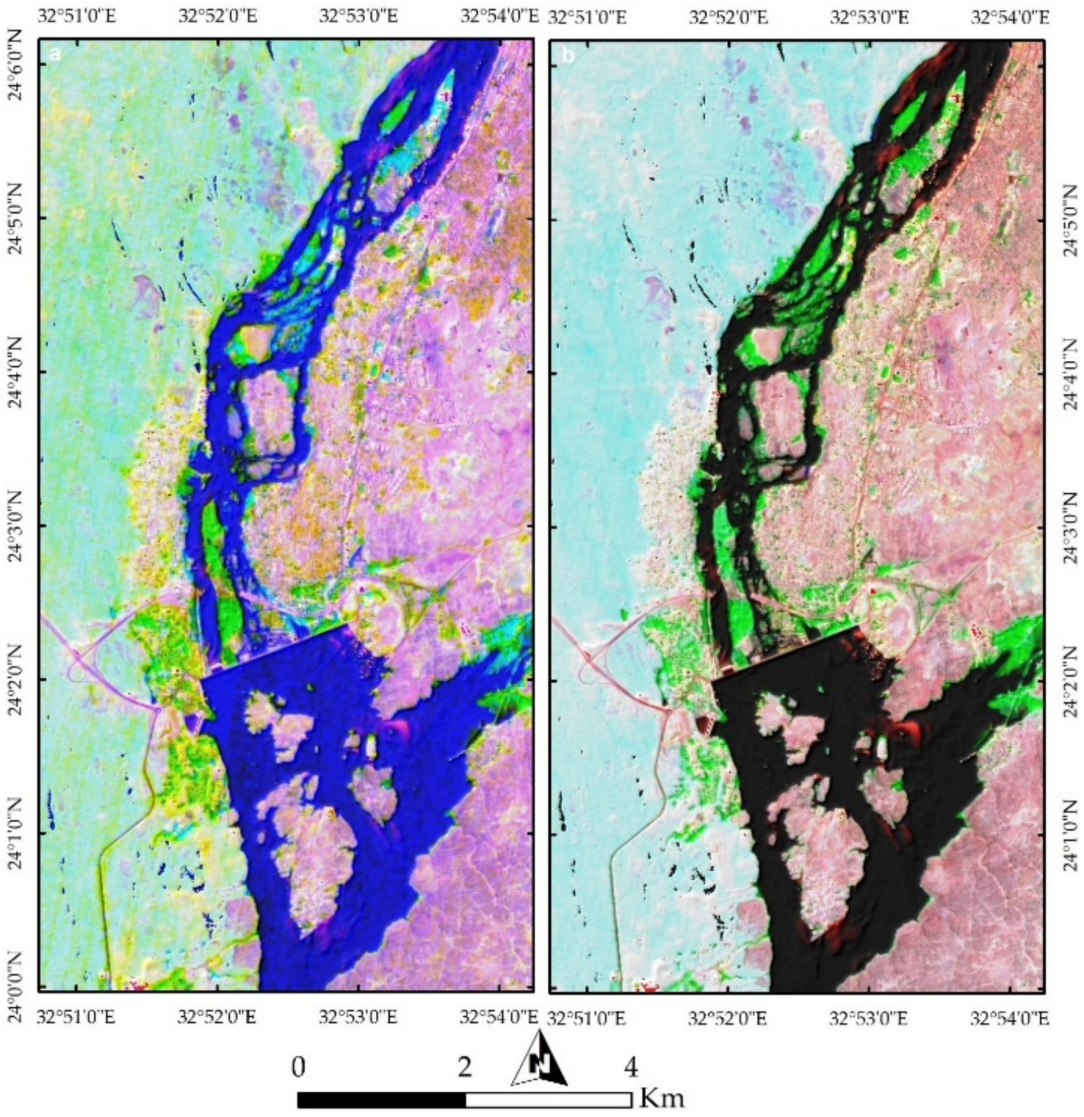
(**a**) PRISMA false color composite (FCC) of bands 37-57-7 in RGB, distinguishing key geological and environmental features of Aswan area. The Nile River, shown in blue, acts as a natural divide between the Nubian Sandstone terrain (cyan) on the west and granitic rocks (pink) on the east. Urban areas appear as yellow clusters. (**b**) FCC of bands 4-78-141, offering enhanced visualization of vegetation cover across agricultural lands and Nile islands.

In this study, PRISMA false color combinations of bands 37-57-7 and FCC 4-78-141 in RGB were utilized to analyze the current geological and environmental setting of the study area. Figure 11a clearly illustrates the Nile River in blue, separating two major terrains: a cyan-colored western area dominated by Nubian Sandstone (NSS) and a pinkish eastern region composed mainly of granitic rocks. The river valley exhibits pronounced curvature and convexity in Nubian Sandstone, highlighting the effects of differential erosion between the granitic rocks and Nubian Sandstone. The water more easily erodes Nubian Sandstone compared to the more resistant granitic rocks. These observations emphasize how geological formations, particularly Nubian Sandstone and granite, influence the river’s curvature and shape the current geomorphology of the study area.

**Fig. 12:**
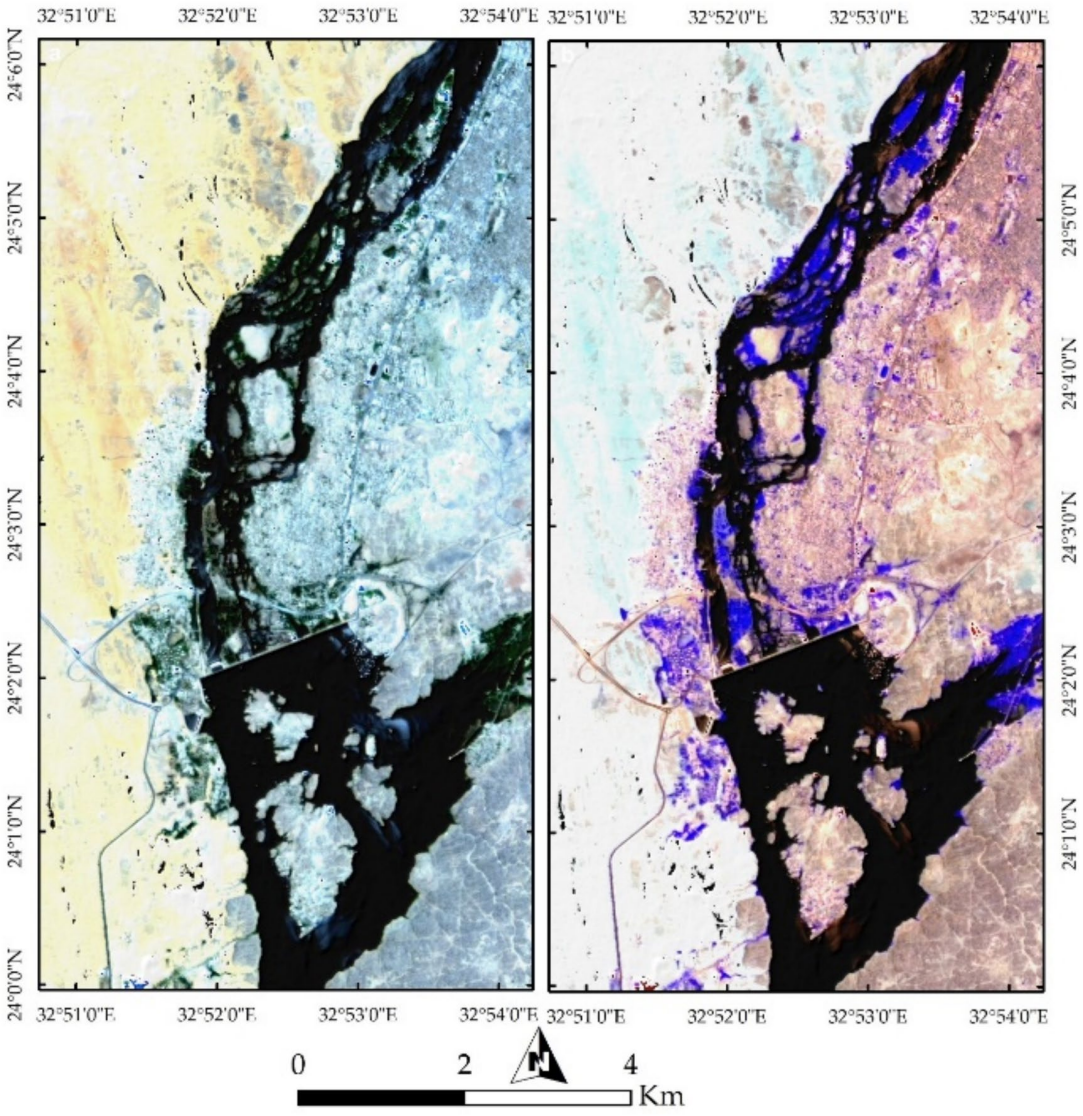
(**a**) FCC of bands 30-20-9 in RGB, enhancing the contrast between Nubian Sandstone and granite formations while highlighting urban encroachment along the riverbanks. (**b**) FCC of bands 18-30-54, refining lithological distinctions and improving the detection of vegetated regions in purple.

Further examination of Fig. 11a reveals buildings represented as yellow dots, mainly concentrated around the Nile River, alongside vegetation appearing in green. Figure 11b provides a clearer depiction of vegetation, represented in green, covering agricultural lands within the city and on the islands in the Nile River. A comparison of previous geological map (Fig. 2) with the current land use and land cover (LULC) highlights the transformation of the natural landscape due to urban development. Figures 12a and 4b, utilizing FCC combinations of 30-20-9 and 18-30-54 in RGB, distinctly differentiate Nubian Sandstone from granite. Figure 12b further emphasizes the natural color separation, with vegetation appearing more distinctly in blue.

**Fig. 13:**
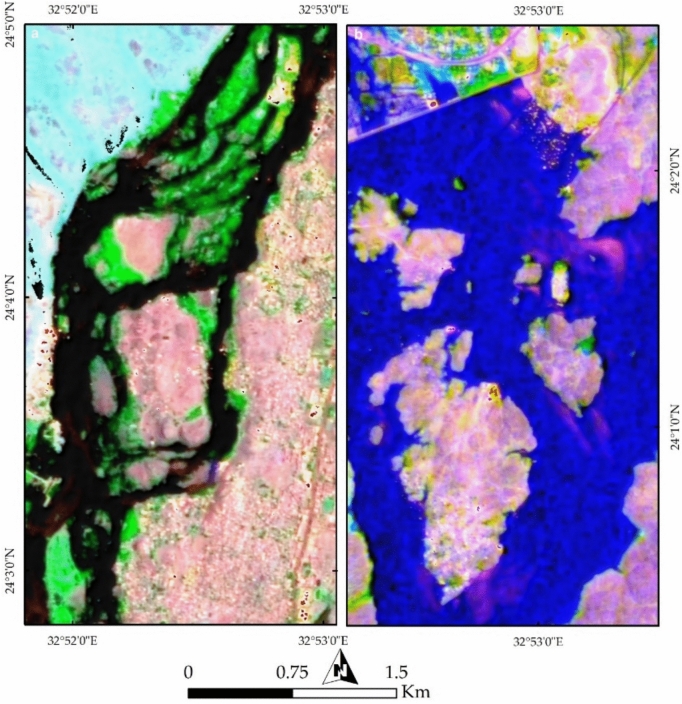
(**a**) Geospatial analysis of Nile islands, including Seheil, Salouga, and Ghazal, illustrating the influence of fluvial processes and structural controls on their morphology. Notable erosion patterns and wadi alignments are observed, particularly along Seheil Island’s southern margin. (**b**) Philae Island’s geomorphological characteristics, revealing how variations in lithological resistance influence erosion, with durable granitic sections remaining intact while weaker gneisses, schists, and Nubian sandstone exhibit greater degradation at the southern tip.

Further confirmation of the geological influence on geomorphology is evident through geospatial analysis of the islands within the Nile River course. Examining Seheil Island and its surroundings (Fig. 13a) reveals the role of water action, weathering, and erosion in shaping these landforms. The southern part of Seheil Island is visibly dissected due to its exposure to water flow, while the Northeastern tip aligns with a wadi course. North of Seheil Island, Salouga and Ghazal islands exhibit a northeastward orientation, marked by deeper erosion along the eastern bank of the Nile, reflecting the action of water. Similarly, the eastern bank of the Nile River within the granitic terrain (at the southern part of the study area) displays angular wadi tracks, indicative of structural control on water pathways.

Analysis of Philae Island (Fig. 13b) in the southern part of the valley further demonstrates the influence of geological resistance on river branching. Philae Island has remained intact due to the durability of its lithology, primarily composed of granitic rocks in its central and northern sections, while its southern tip, consisting mainly of gneisses, schists, and Nubian Sandstone, appears more eroded. This differential erosion has played a crucial role in shaping both the island and the branching of the Nile River.

#### Environmental and Anthropogenic Influences

**Fig. 14:**
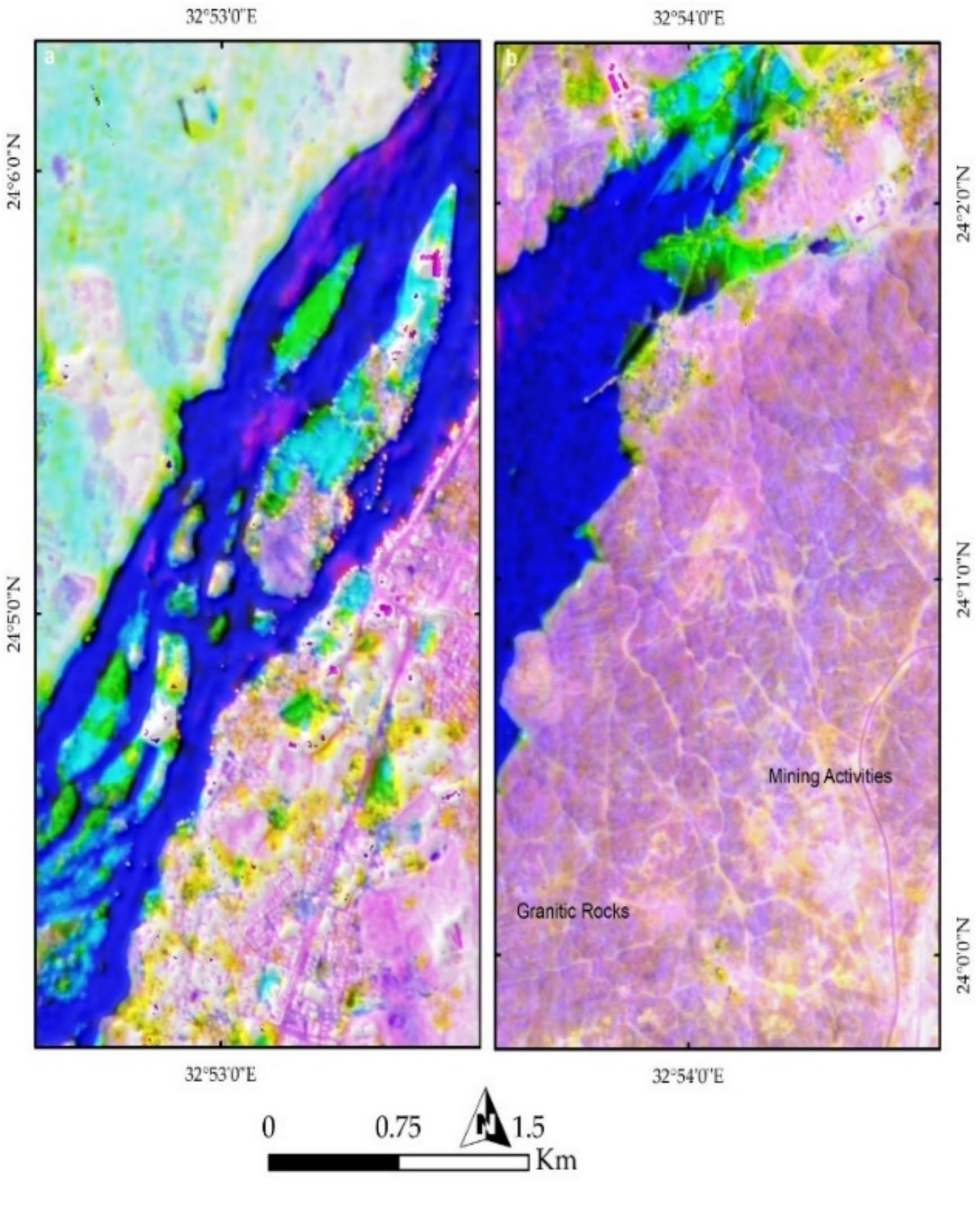
(**a**) Spatial distribution of Aswan’s islands along the NNE-SSW axis, shaped by geological structures and hydrodynamic forces of the Nile. Urban settlements, marked in yellow, are concentrated along the riverbanks, while bright pixels indicate active quarry-mining operations at the southeastern corner. (**b**) FCC visualization emphasizing quarrying and extraction sites (bright and yellow pixels) within granitic terrains, highlighting their proximity to expanding urban infrastructure.

**Fig. 15:**
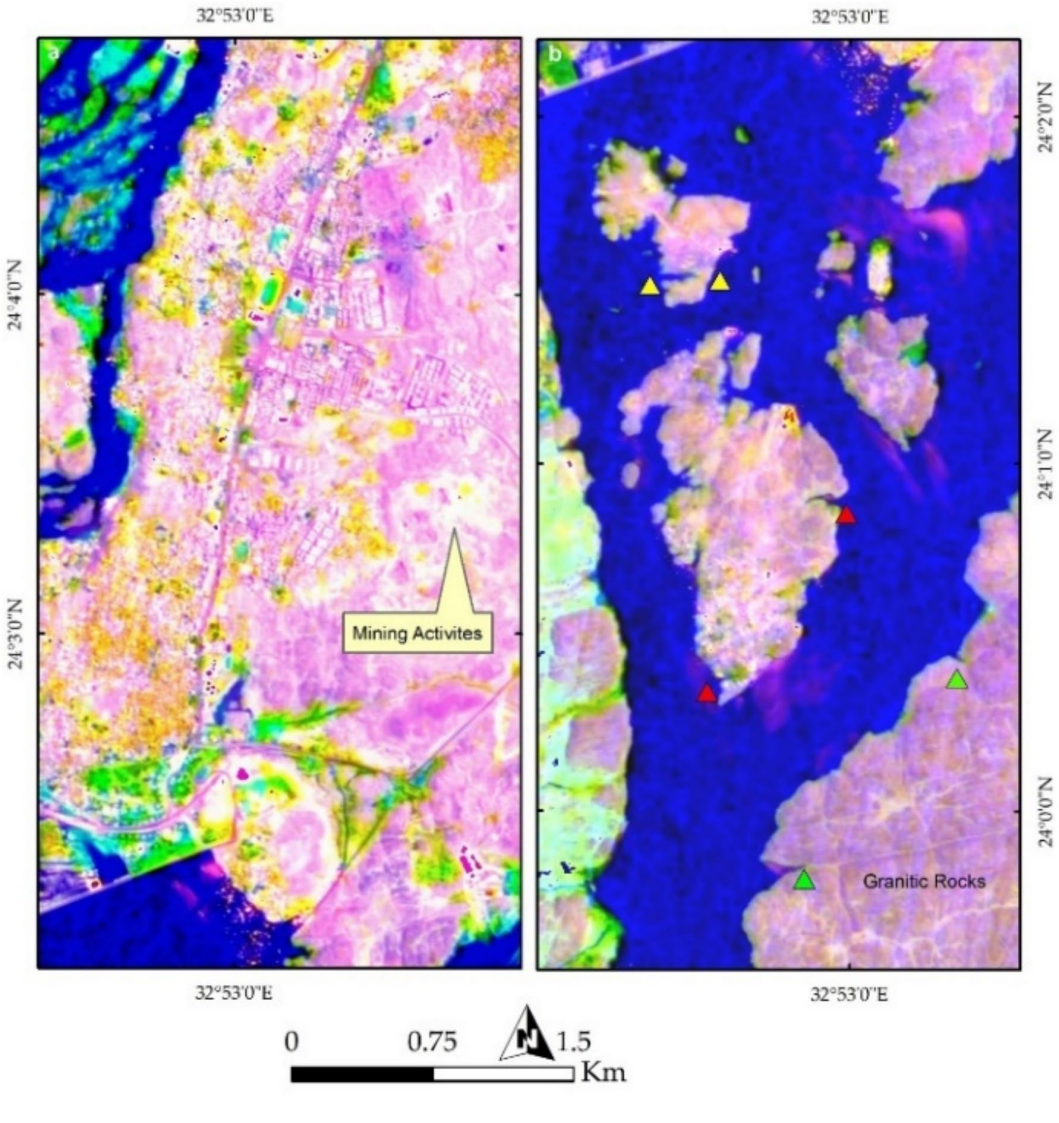
(**a**) Remote sensing analysis detecting quarrying activities and their landscape impact, demonstrating the extent of land modification due to industrial operations. (**b**) Predicted patterns of future island fragmentation based on ongoing fluvial erosion, indicating vulnerable areas within the southern sector of the study region.

The spatial arrangement of Aswan’s islands and their alignment along the NNE-SSW trend reflects the interplay between the Nile River and underlying rocks (Fig. 14a). Anthropogenic activities are prominently visible in yellow, representing buildings, while bright pixels in the southeastern portion indicate active mining operations. Figures 14b and 7a provides a clearer visualization of mining and quarry activities within the granitic rocks, revealing their proximity to urban settlements. Figure 15b highlights potential areas of future dissection within the southern part of the study area, illustrating how island fragmentation may progress over time.

#### Geomorphological Implications

**Fig. 16:**
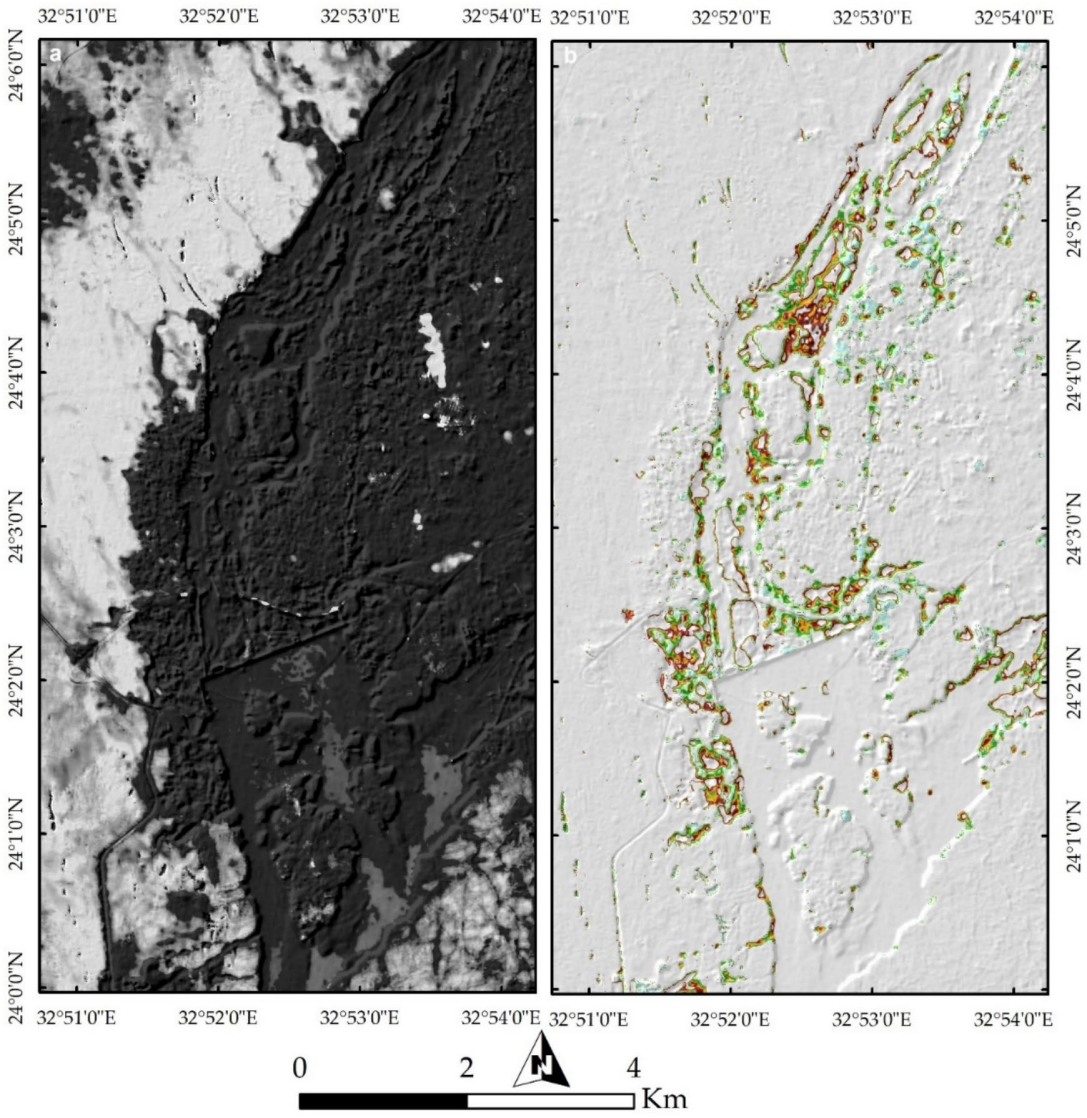
(**a**) Endmember analysis mapping preserved natural geological formations (white) and highly altered regions due to human activity (black). (**b**) SMACC Endmember 7 identifying zones of active erosion, particularly around Philae Island and along major sections of the Nile River valley.

Figure 16a (Endmember 6) highlights remaining natural lithological landscapes in white, predominantly representing the western Nubian Sandstone terrain and the southeastern granitic block. The black-colored areas in Fig. 8a represent regions heavily affected by anthropogenic activities. Further analysis using SMACC Endmember 7 emphasizes (Fig. 16b) the impact of the Nile River, with colored pixels marking areas highly vulnerable to further erosion and dissection, particularly along Philae Island and the broader valley course.

**Fig. 17:**
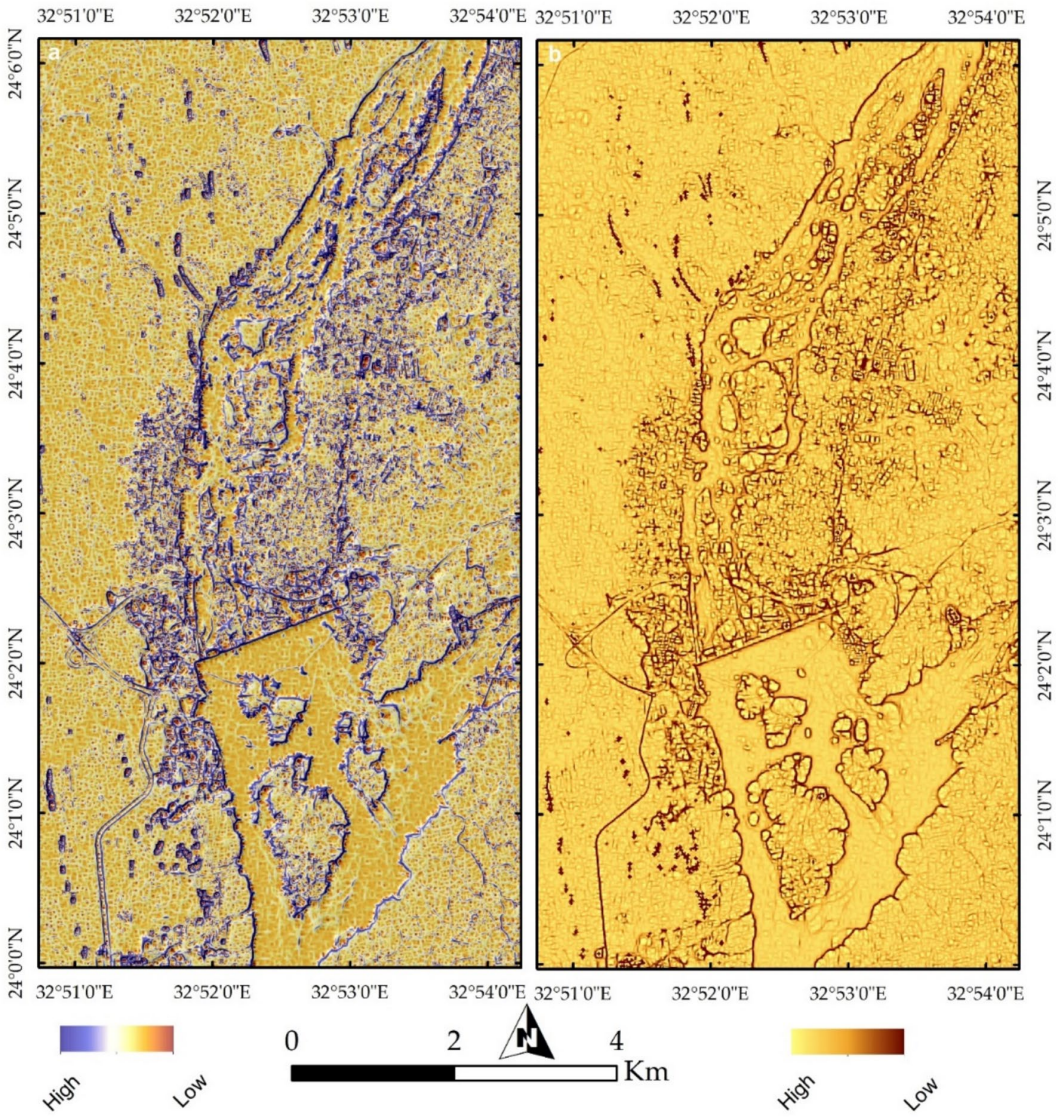
(**a**) Maximum and (**b**) minimum curvature topographic images, capturing geomorphological variations and anthropogenic modifications such as building edges, infrastructure, and landscape alterations.

**Fig. 18:**
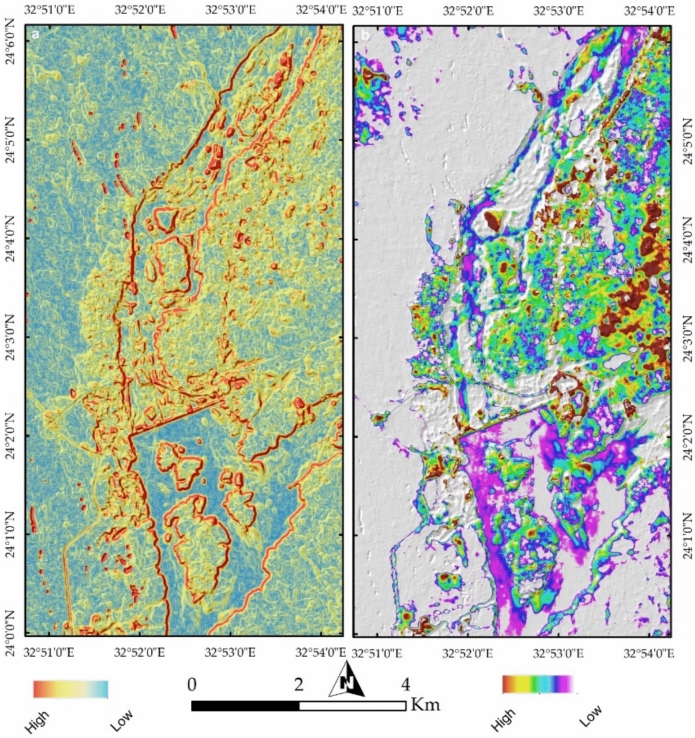
(**a**) Slope analysis delineating steep escarpments along the Nile Valley and island peripheries, with red-marked zones indicating areas susceptible to future erosion. (**b**) Endmember 8 visualization illustrating lithological variability, urban expansion, and surface heterogeneity across the region.

The influence of urbanization and industrial activities is further assessed through maximum and minimum curvature topographic images (Fig. 17a,b), which highlight anthropogenic features and building edges. The slope map (Fig. 18a) delineates the boundaries of the Nile Valley cliffs and island margins in red, marking potential future erosion zones in red troughs, particularly around Philae Island and along the entire river course. Notable heterogeneity in lithology, infrastructure, and even inside the water body is also observed in Fig. 18b through Endmember 8.

**Fig. 19:**
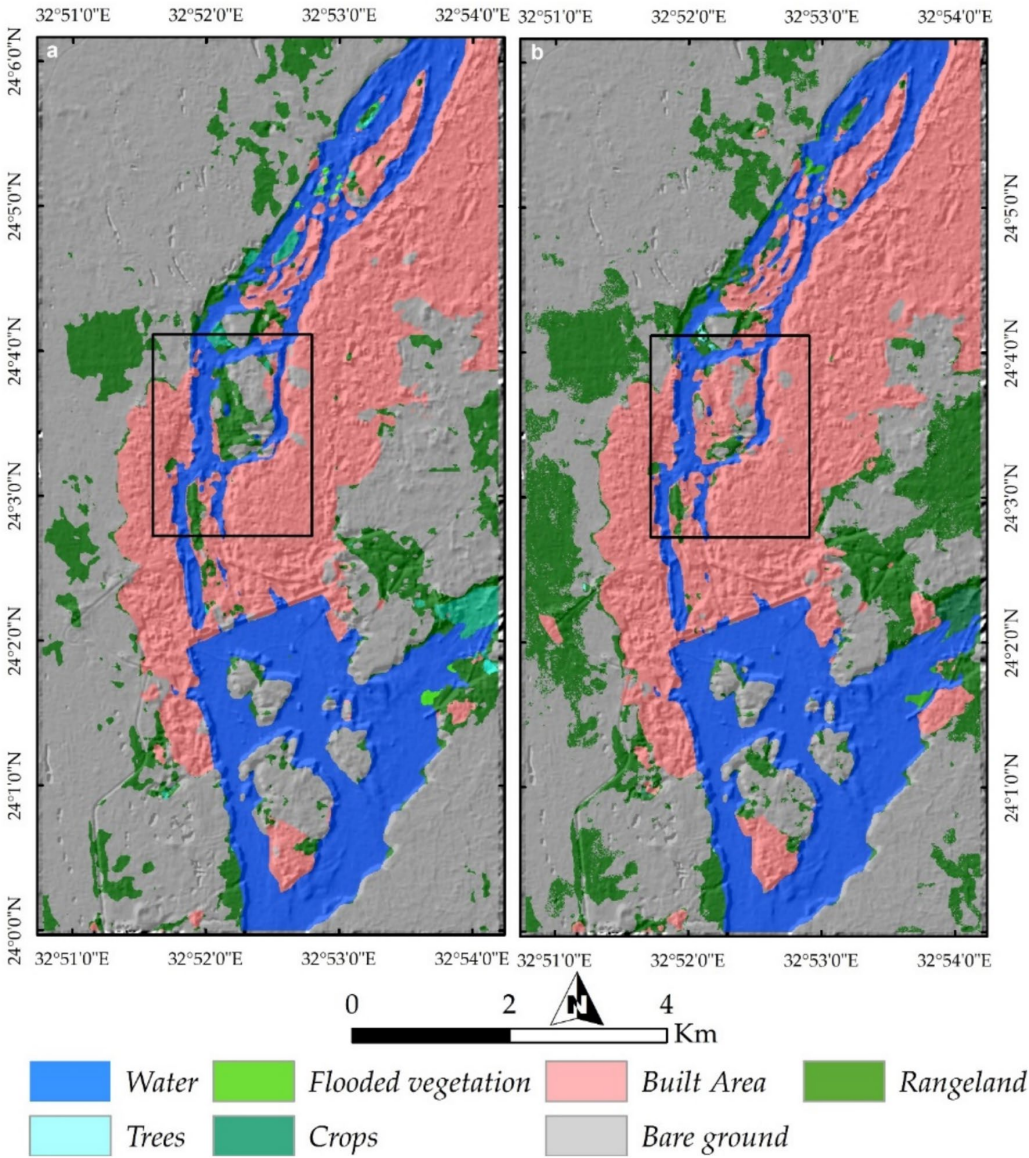
(**a**, **b**) Land-use and land-cover (LULC) analysis derived from Sentinel-2 data, comparing spatial changes between 2017 and 2023. Black rectangles highlight significant urban expansion on Seheil Island and increased vegetation cover on Philae Island, reflecting shifts in land management and ecological dynamics.

An ESRI LULC map based on Sentinel-2 data provides insight into land-use changes over the last six years (Fig. 19a,b). A comparison of these maps from 2017 to 2023 highlights significant transformations, including the expansion of rangeland on Philae Island and increased urban development on Seheil Island, as indicated by black rectangles on the maps.

### Radiometric results

#### Activity Concentrations and radiation hazard indices

**Fig. 20:**
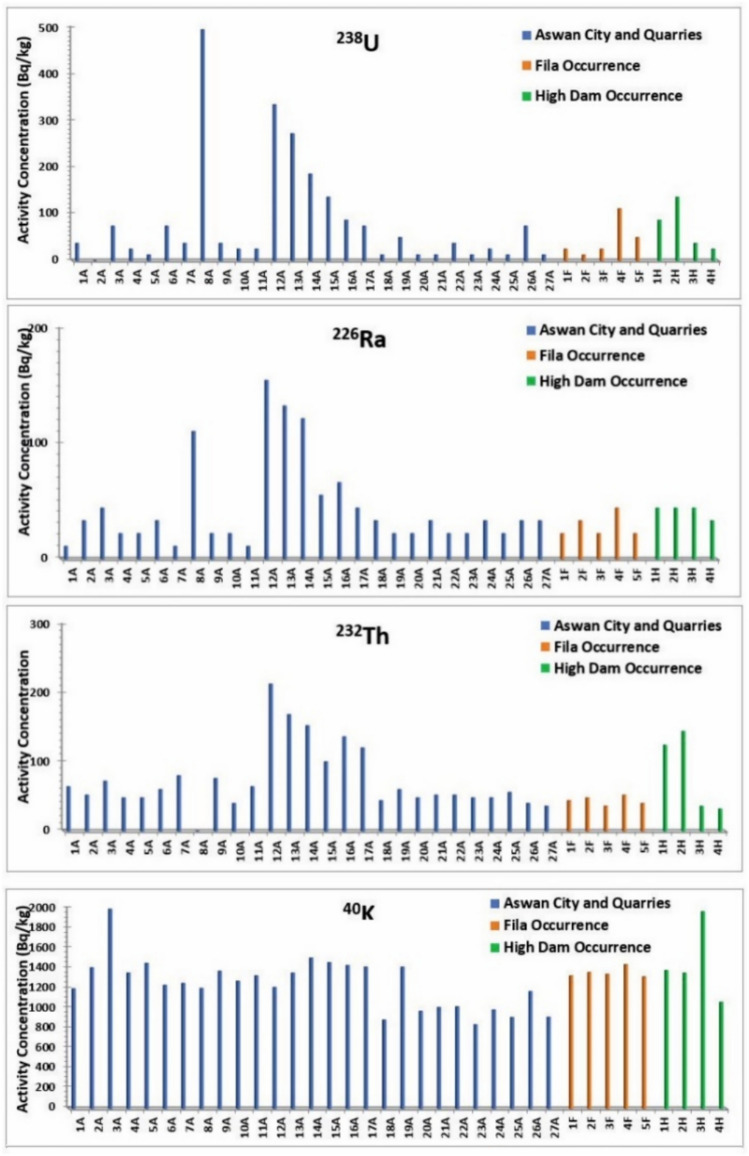
Activity concentrations (Bq/kg) of ^238^U, ^226^Ra, ^232^Th, and ^40^K in the analyzed samples.

The activity concentration of the natural radionuclides ^238^U, ^226^Ra, ^232^Th and ^40^K in the analyzed granite samples are summarized in Table 1. The results indicate notable spatial variations in radionuclide concentrations across the studied occurrences. As illustrated in Fig. 20, granite samples collected from Aswan City and adjacent quarry sites exhibit a wide range of activity levels. The ^238^U activity concentrations vary between 12.4 and 496 Bq/kg, while ^226^Ra ranges from 11.1 to 155.4 Bq/kg. The ^232^Th activity concentrations range from < 0.6 to 214.12 Bq/kg, with corresponding average values of 84.42, 44.4, and 76.76 Bq/kg. The ^40^K activity concentrations display values between 838.84 and 1993.81 Bq/kg, with an overall mean of 1243.31 Bq/kg. In the Fila occurrence, the ^238^U activity concentrations range from 12.4 to 111.6 Bq/kg, while ^226^Ra ranges between 22.2 and 44.4 Bq/kg, with average values of 44.64 and 28.86 Bq/kg, respectively. The ^232^Th activity concentrations range from 36.36 to 52.52 Bq/kg, yielding an average value of 44.44 Bq/kg. The ^40^K activity concentrations range from 1317.73 to 1439.8 Bq/kg, with a mean value of 1357.79 Bq/kg. In the High Dam occurrence, ^238^U activity concentrations range from 24.8 to 136.4 Bq/kg, whereas ^226^Ra ranges between 33.3 and 44.4 Bq/kg, with average values of 71.3 and 41.62 Bq/kg, respectively. The ^232^Th activity concentrations vary from 32.32 to 145.44 Bq/kg, with an average value of 84.84 Bq/kg. The ^40^K concentrations range between 1064.2 and 1968.77 Bq/kg, with an average of 1442.15 Bq/kg.

**Table 1:** The activity concentrations (Bq/kg) of ^238^U, ^226^Ra, ^232^Th and ^40^K for the samples studied.

Sample location	Sample	^238^U (Bq/kg)	^226^Ra (Bq/kg)	^232^Th (Bq/kg)	^40^K (Bq/kg)
Aswan City and Quarries	Coarse pink granites (Syenogranites and monzogranites)				
	1A	37.20	11.10	64.64	1198.79
	2A	24,80	33.30	52.52	1408.50
	3A	74.40	44.40	72.72	1993.81
	4A	24.80	22.20	48.48	1355.29
	5A	12.40	22.20	48.48	1452.32
	6A	74.40	33.30	60.60	1233.22
	7A	37.20	11.10	80.80	1252.00
	8A	496.00	111.00	< 0.6	1201.92
	9A	37.20	22.20	76.76	1374.07
	10A	24.80	22.20	40.40	1273.91
	11A	24.80	11.10	64.64	1327.12
	Fine granites (Monzogranites)				
	12A	334.80	155.40	214.12	1211.31
	13A	272.80	133.20	169.68	1355.29
	14A	186.00	122.10	153.52	1505.53
	15A	136.40	55.50	101.00	1458.58
	16A	86.80	66.60	137.36	1430.41
	17A	74.40	44.40	121.20	1414.76
	High Dam granites (gneissose granodiorites, sheared monzogranites)				
	18A	12.40	33.30	44.44	885.79
	19A	49.60	22.20	60.60	1414.76
	20A	12.40	22.20	48.48	973.43
	21A	12.40	33.30	52.52	1010.99
	22A	37.20	22.20	52.52	1017.25
	23A	12.40	22.20	48.48	838.84
	24A	24.80	33.30	48.48	985.95
	25A	12.40	22.20	56.56	910.83
	26A	74.40	33.30	40.40	1170.62
	27A	12.40	33.30	36.36	913.96
	Average	84.42	44.40	76.76	1243.31
Fila Occurrence	Fila coarse pink granites (Syenogranites)				
	1F	24.80	22.20	44.44	1327.12
	2F	12.40	33.30	48.48	1361.55
	3F	24.80	22.20	36.36	1342.77
	4F	111.60	44.40	52.52	1439.80
	5F	49.60	22.20	40.40	1317.73
	Average	44.64	28.86	44.44	1357.79
High Dam Occurrence	Greyish pink (dark red) (Granodiorites)				
	1H	86.80	44.40	125.24	1380.33
	2H	136.40	44.40	145.44	1355.29
	3H	37.20	44.40	36.36	1968.77
	4H	24.80	33.30	32.32	1064.20
	Average	71.3	41.625	84.84	1442.15
Recommended Values		35	35	30	400

The minimum and maximum values of activity concentrations for ^238^U, ^232^Th and ^40^K in all studied samples are 77.23, 41.93, 73.07, and 1281.3 Bq/kg, respectively (Table 1; Fig. 20), which are relatively higher than the world average levels of 35, 35, 30, and 400 Bq/kg, respectively^25^. Among the radionuclides, potassium exhibits the highest activity concentration in all samples and exceeds the recommended value of 400 Bq/kg. This elevated level is attributed to the presence of potassium feldspar and sodium feldspar in granites, consistent with the fact that potassium in the Earth’s crust occurs at percent levels, whereas uranium and thorium are present at ppm levels^26^. The minimum and maximum values of activity concentrations 0f. ^238^U, ^232^Th and ^40^K were observed in samples from Aswan city and quarries, while for ^226^Ra, the minimum and maximum values were found in Aswan city & quarries and High Dam occurrence, respectively.

From Table 1, it is notable that the mean activity concentrations follow specific trends by location:

- ^238^U and ^226^Ra: Aswan city & quarries > High Dam occurrence > Fila occurrence
- ^232^Th: High Dam occurrence > Aswan city & quarries > Fila occurrence
- ^40^ K: High Dam occurrence > Fila occurrence > Aswan city & quarries

These trends reflect element-specific geochemical distributions, with uranium and radium being highest in Aswan city and quarries, thorium in High Dam occurrence, and potassium highest in High Dam occurrence. The calculated values of radium equivalent activity (Ra_eq_), internal and external hazard indices (H_in_ and H_ex_), and the representative gamma level index (I_γ_) for the studied granite samples are presented in Table 2. These parameters were used to assess the potential radiological risks associated with the use of these rocks for construction and other purposes.

**Table 2:** The calculated values of absorbed dose rate (D), the annual effective dose equivalent (AEDE), radium equivalent activity (Ra_eq_), internal hazard index (H_in_), external hazard index (H_ex_) and radiation level index (I_γ_) of the samples under investigation.

Sample location	Sample	D (nGy/h)	*AEGD *(mSv/y)	Ra_eq_ (Bq/kg)	*H*_*in*_	*H*_*ex*_	*I*_*γ*_
Aswan City and Quarries	Coarse pink granites (Syenogranites and monzogranites)						
	1A	106.22	0.13	221.76	0.60	0.70	1.69
	2A	101.91	0.12	208.17	0.56	0.63	1.63
	3A	161.44	0.20	331.66	0.90	1.10	2.55
	4A	97.26	0.12	198.31	0.54	0.60	1.55
	5A	95.57	0.12	193.37	0.52	0.56	1.54
	6A	122.40	0.15	255.83	0.69	0.89	1.92
	7A	118.20	0.14	248.94	0.67	0.77	1.89
	8A	279.27	0.34	588.46	1.59	2.93	4.11
	9A	120.85	0.15	252.55	0.68	0.78	1.93
	10A	88.98	0.11	180.51	0.49	0.55	1.42
	11A	105.84	0.13	219.23	0.59	0.66	1.70
	Fine granites (Monzogranites-syenogranite)						
	12A	334.52	0.41	733.86	1.98	2.89	5.18
	13A	285.04	0.35	619.45	1.67	2.41	4.42
	14A	241.44	0.30	521.12	1.41	1.91	3.78
	15A	184.84	0.23	392.88	1.06	1.43	2.89
	16A	182.72	0.22	393.06	1.06	1.30	2.91
	17A	166.57	0.20	356.37	0.96	1.16	2.65
	Grey granites (Granodiorites-tonalites, quartz diorites)						
	18A	69.51	0.09	144.02	0.39	0.42	1.12
	19A	118.51	0.15	245.00	0.66	0.80	1.88
	20A	75.60	0.09	156.54	0.42	0.46	1.22
	21A	79.61	0.10	165.20	0.45	0.48	1.28
	22A	91.33	0.11	190.48	0.51	0.62	1.45
	23A	69.99	0.09	146.18	0.40	0.43	1.13
	24A	81.85	0.10	169.90	0.46	0.53	1.31
	25A	77.87	0.10	163.26	0.44	0.47	1.26
	26A	107.59	0.13	222.16	0.60	0.80	1.68
	27A	65.80	0.08	134.65	0.36	0.40	1.06
	Average	134.47	0.17	283.44	0.77	0.99	2.12
Fila Occurrence	Fila coarse pink granites (Syenogranites)						
	1F	93.64	0.11	190.37	0.51	0.58	1.49
	2F	91.79	0.11	186.39	0.50	0.54	1.48
	3F	89.41	0.11	180.03	0.49	0.55	1.42
	4F	143.32	0.18	297.38	0.80	1.11	2.23
	5F	102.27	0.13	208.68	0.56	0.70	1.61
	Average	104.09	0.13	212.57	0.57	0.70	1.65
High Dam Occurrence	Greyish pink (Granodiorites)						
	1H	173.31	0.21	371.89	1.01	1.24	2.75
	2H	207.38	0.25	448.42	1.21	1.58	3.27
	3H	121.25	0.15	240.59	0.65	0.75	1.92
	4H	75.36	0.09	152.83	0.41	0.48	1.20
	Average	144.33	0.18	303.43	0.82	1.01	2.29
Recommended Values		57	0.48	370	1	1	1

**Fig. 21:**
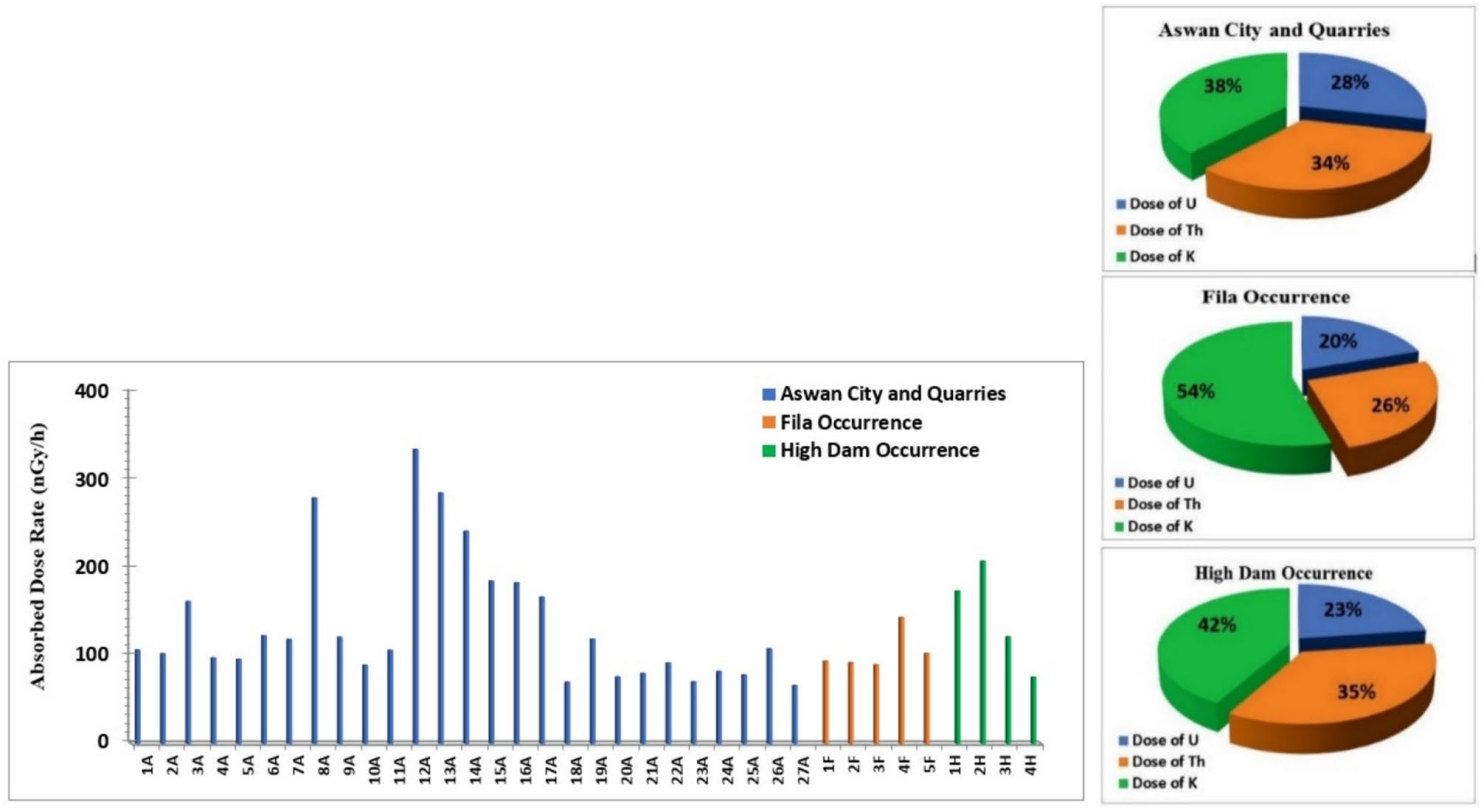
(**a**) Absorbed dose rate (nGy/h) for the analyzed samples; (**b**) Contributions of individual radionuclides to the absorbed gamma dose rate in the analyzed samples.

The absorbed dose rate (D) in Aswan City and quarry samples varies from 65.8 to 334.52 nGy/h, with an average value of 134.47 nGy/h. In the Fila occurrence, the absorbed dose rate ranges from 89.41 to 143.32 nGy/h, with an average of 104.09 nGy/h, while in the High Dam occurrence, values range between 75.36 and 207.38 nGy/h, yielding an average of 144.33 nGy/h. All measured absorbed dose rate values exceed the internationally permissible level (Fig. 21a). Overall, the absorbed dose rate (D) for all studied samples ranges between 65.8 and 334.52 nGy/h, with an overall mean of 131.35 nGy/h, which is significantly higher than the global average of approximately 57 nGy/h (UNSCEAR, 2000). The relative contributions of ^238^U, ^232^Th, and ^40^K to the total absorbed gamma dose rate for all studied samples are illustrated in Fig. 21b.

**Fig. 22:**
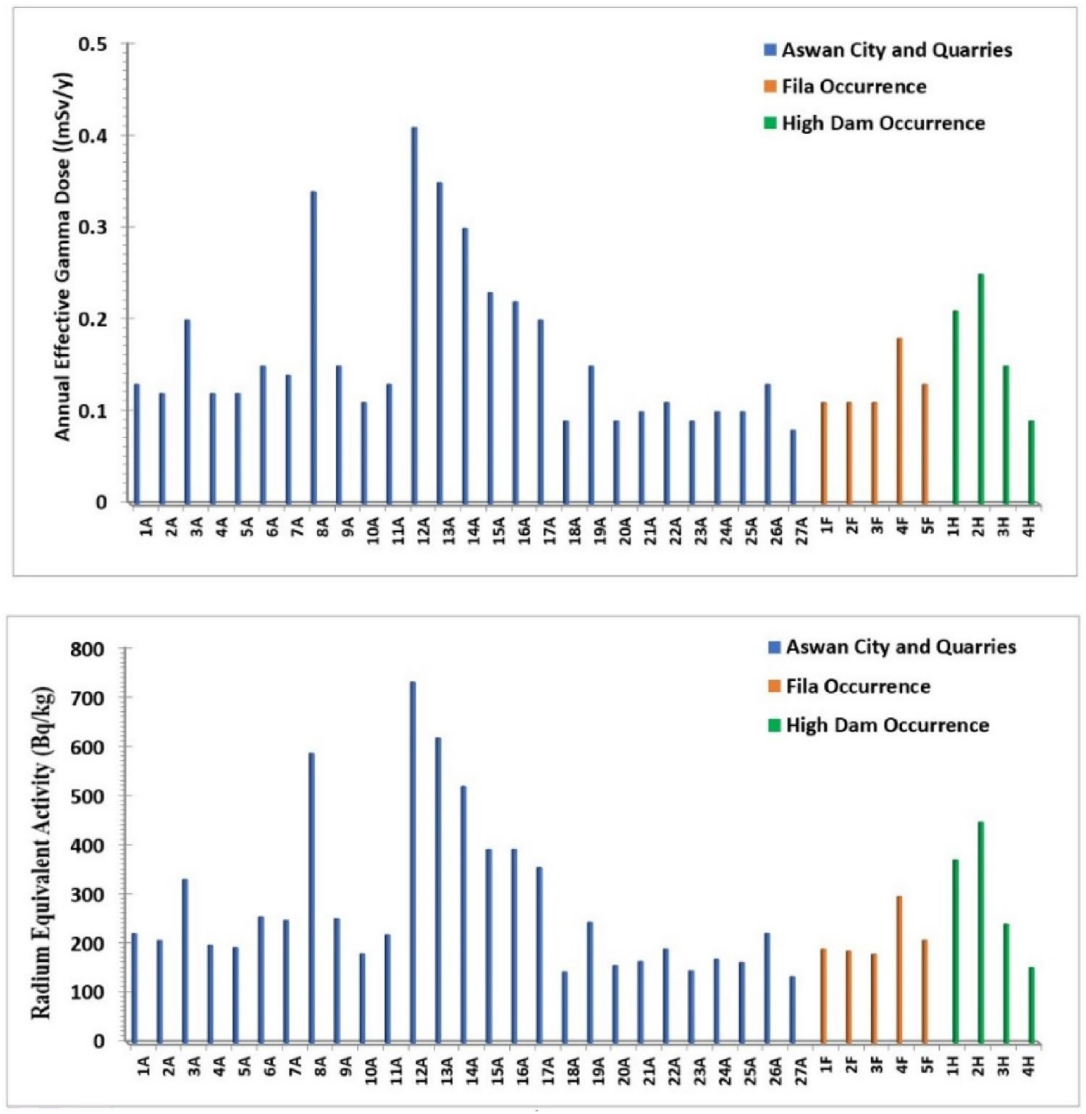
(**a**) Annual effective gamma dose (mSv/y) for the analyzed samples; (**b**) Radium equivalent activity (Bq/kg) in the analyzed samples.

Natural radiation is the largest contributor to the external dose received by the world population, primarily due to gamma radiation from different geological settings^21^. The gamma dose rates vary geographically depending on the concentrations of natural radionuclides such as ^238^U, ^232^Th and their progenies, as well as the activity of singly occurring radionuclides like ^40^ K. The annual effective gamma dose (AEGD) for all investigated samples ranges from 0.09 to 0.41 mSv/y, with an average of 0.16 mSv/y (Fig. 22a). All values are below the worldwide average recommended dose of 0.48 mSv/y^21^.

The radium equivalent activity (Ra_eq_) for samples from Aswan city and quarries ranges from 134.65 to 733.86 Bq/kg, with an average of 283.44 Bq/kg, while samples from Fila occurrence range between 180.03 and 297.38 Bq/kg, with an average of 212.57 Bq/kg. For High Dam occurrence, Ra_eq_ varies from 152.83 to 448.42 Bq/kg, with an average of 303.43 Bq/kg. Considering all samples together, (Ra_eq_) ranges from 134.65 to 733.86 Bq/kg, with an overall average of 275.82 Bq/kg. As illustrated in Fig. 22b, all radium equivalent values are below the permissible limit except for eight samples (8A, 12A, 13A, 14A, 15A, 16A, 1H, and 2H).

**Fig. 23:**
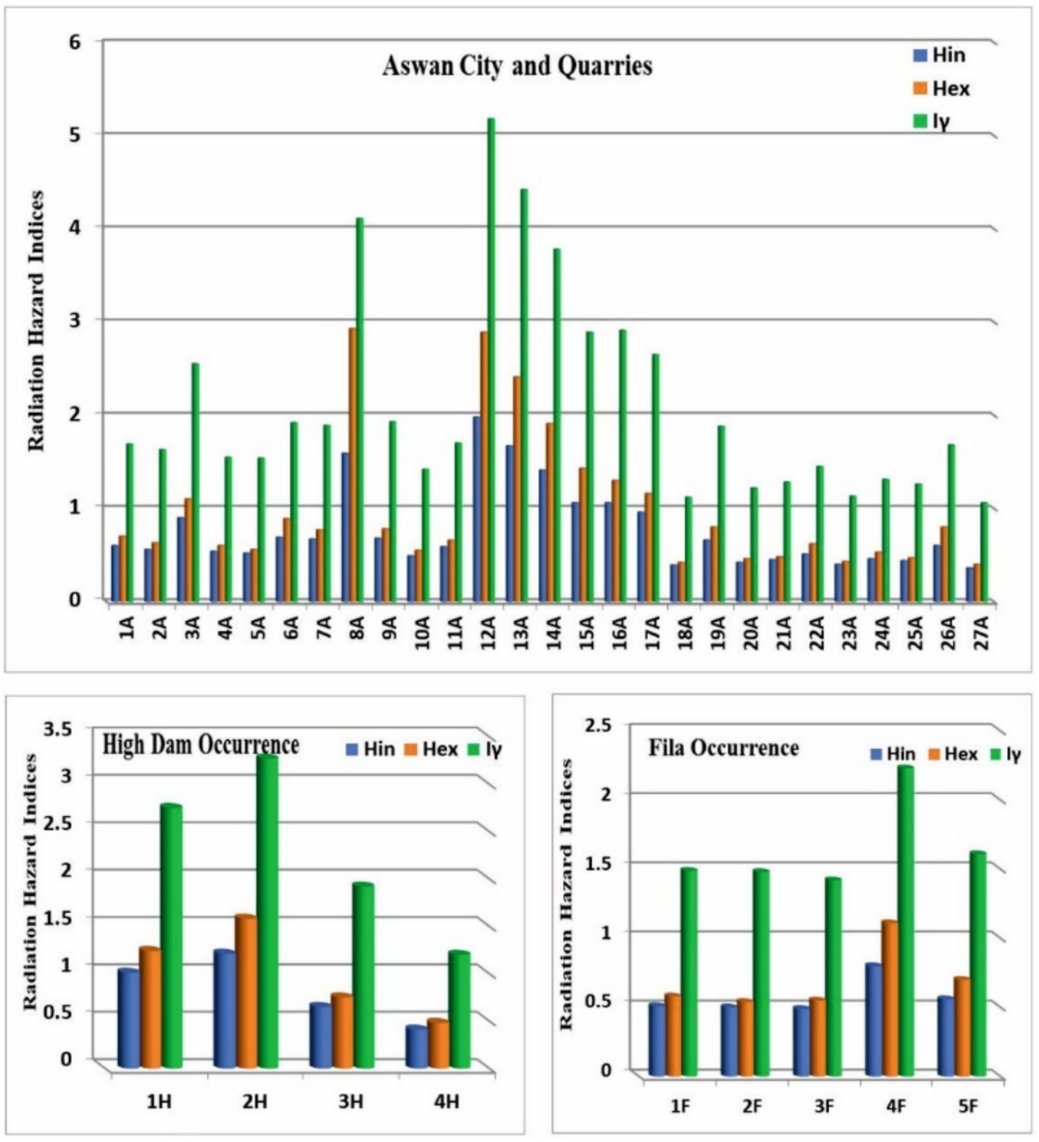
Internal hazard index (H_in_), external hazard index (H_ex_) and gamma index (I_γ_) for all analyzed sample types.

The internal and external hazard indices (H_in_ and H_ex_) and the gamma radiation index (I_γ_) for the granitic samples are presented in Fig. 23. Except for eight samples, all H_in_ values are below the permissible level, and except for eleven samples, all H_ex_ values are below the permissible level. The average values of the internal and external hazard indices are 0.74 and 0.95, respectively, both below unity. In contrast, all values of the gamma radiation index (I_γ_) exceed the permissible level of unity.

## Discussion

Field observations integrated with petrographic analysis, geomorphological mapping, remote sensing, and radiological measurements provide a comprehensive understanding of the Aswan granite province and its geological and environmental significance. This region represents one of Egypt’s most important Neoproterozoic granite terrains, where diverse granitoid lithologies, intrusive relationships, structural frameworks, geomorphological expressions, and radiological characteristics record a prolonged, multi-stage magmatic evolution overprinted by tectonic deformation and weathering processes that collectively shaped the present Aswan landscape.

### Geological controls on Aswan granite landscapes

He Aswan granitoids comprise four main types with distinct geological and petrographic characteristics. Greyish-black tonalites–granodiorites are early intrusions with coarse porphyritic textures dominated by quartz, plagioclase, K-feldspar, and biotite–amphibole, forming broad pediments and moderate slopes. Coarse pink monzogranites–syenogranites intruded later, featuring K-feldspar megacrysts, perthite, myrmekite, and accessory zircon–apatite–sphene, producing rugged, blocky terrain, Nile islands, and spheroidal weathering patterns. High-Dam granites, regionally extensive and locally sheared, show recrystallization, mineral banding, and mylonitic fabrics, with mixed pink–grey coloration and medium-to-coarse grains, generating variable weathering profiles and elevated radiological signatures in deformed zones. Fine-grained monzogranites–syenogranites are late-stage intrusions with uniform equigranular textures, aligned biotite, and enriched accessory minerals (zircon–monazite–allanite), forming narrow ridges and fractured bodies prone to granular disintegration and high radiological output. Sequential intrusion from mafic-rich tonalites to K-feldspar–rich evolved granites, controlled by dominant N–S joints and NE-trending shear zones, reflects progressive magmatic differentiation, emplacement dynamics, and subsequent weathering across the Aswan granite province.

Granitic terrains exhibit distinctive landforms governed by rock properties and their spatial variation, with the geometry of the intrusive bodies exerting a strong control on overall landscape morphology. Various approaches to classifying granite landscapes have been proposed in the literature^27^. In the Aswan region, geomorphology is primarily controlled by lithology and structural fabric, with granitic bedrock shaping erosional and weathering-driven landforms. Field observations reveal distinct relief types, including domed and castellated inselbergs, tors, nubbins and granite boulders. Castellated residuals form through deep differential weathering along orthogonal joint systems, while nubbin dome-shaped hills scattered with blocks result from the disintegration of the dome’s outer shell along concentric sheet joints (e.g.,^28^). Granite boulders similarly originate from spheroidal weathering, where water infiltrates joints in massive granite and weathers it in concentric layers (rinds), leaving rounded, relatively unweathered cores known as corestones.

Weathering is a fundamental driver of Earth-surface processes, landform development, and long-term landscape evolution. In the Aswan granites, prolonged physical weathering has produced spheroidal features such as rounded boulders, onion-like exfoliation, and weathering pits; progressive alteration forms corestones encased in concentric shells within decomposed regolith, while spall removal and erosion promote inward weathering and gradual boulder size reduction. Chemical weathering of the granite islands and walls along the river flanks produces weathered surfaces exposed to the river’s annual flooding, with some islands being completely or heavily weathered. This process is primarily controlled by mineral stability, with quartz remaining the most resistant phase, whereas Ca-rich plagioclase, amphiboles, and biotite are more susceptible to alteration through feldspar hydrolysis, leading to the formation of clay minerals such as kaolinite.

Weathering styles vary systematically with granite type and structure. Greyish-black tonalite–granodiorites form broad pediments and moderate slopes, while coarse pink granites dominate rugged, blocky terrains and Nile islands, where abundant quartz, K-feldspar megacrysts, mafic enclaves, and pervasive N–S jointing favor spheroidal weathering. Fine-grained granites form narrow ridges and steep slopes and commonly undergo granular disintegration, whereas High-Dam granites exhibit variable weathering related to textural heterogeneity and localized NE-trending shear zones, with undeformed domains resembling coarse pink granites and mylonitized zones developing gullies. Along the Nile and adjacent islands (e.g., Seheil, Saluja, and Philae), sustained water–rock interaction enhances chemical alteration at lower margins, producing asymmetric slopes characterized by mechanically disintegrated upper zones and chemically weakened bases. Collectively, the interaction of lithology, grain size, jointing, and fluvial processes has generated tors, exfoliation domes, blocky terrains, and smoother slopes, defining the rugged, island-studded geomorphology of the Aswan granite province.

### Assessing Aswan Granites via Remote Sensing and Radiological Analysis

PRISMA hyperspectral imagery provides a comprehensive framework for interpreting the geological architecture, geomorphology, and anthropogenic modifications of the Aswan area. False color composites (FCC 37–57–7 and 4–78–141) clearly discriminate the Nubian Sandstone–dominated western sector (cyan tones) from the granitic terrain along the eastern Nile bank (pinkish hues), reflecting contrasting mineralogy and spectral responses (e.g.^29^). This lithological contrast exerts a first-order control on Nile River morphology, with pronounced curvature over Nubian Sandstone and a confined course through resistant granites, demonstrating the role of differential erosion in shaping the Aswan Valley. Vegetation and urban patterns further emphasize the coupling between geology, geomorphology, and human activity, with vegetation concentrated on floodplains and islands, and built-up areas clustered along riverbanks. Additional FCC combinations (30–20–9; 18–30–54) enhance granite–sandstone separation and vegetation discrimination, highlighting PRISMA’s ability to resolve subtle lithological and environmental variations beyond conventional multispectral imagery.

Remote sensing of Nile islands reveals lithology-structure-fluvial process interactions. Seheil Island shows differential erosion and wadi alignment; Salouga and Ghazal islands are structurally oriented NE; and Philae Island demonstrates lithological control on river branching, with resistant granites maintaining morphology and softer rocks eroded. Anthropogenic impacts are also evident: quarrying within coarse pink and High-Dam granites appears as sharp, high-reflectance surfaces, stepped quarry faces, waste dumps, and modified slopes. Endmember and curvature analyses delineate undisturbed lithologies, fluvially influenced zones, and human-modified landscapes, while slope analysis identifies potential erosion hotspots. Temporal Sentinel-2 LULC mapping (2017–2023) documents urban expansion, rangeland growth, and intensified quarrying, showing that anthropogenic activity increasingly dominates landscape modification. PRISMA imagery also discriminates the four granite suites: greyish-black tonalites–granodiorites (dark spectral signatures), coarse pink monzogranites–syenogranites and High-Dam granites (pink-red reflectance), and fine-grained granites (intermediate signatures), consistent with field petrography.

Although naturally occurring radionuclides in granitic rocks elevate background radiation, these rocks are widely used as construction materials worldwide, making it essential to assess potential radiological risks to human health and quantify their levels^30,31^. Understanding their concentrations and spatial distribution is crucial for evaluating environmental impacts, and radiological measurements of Aswan granites reveal significant variations associated with lithology and magmatic evolution, providing baseline data for monitoring temporal changes and assessing potential environmental effects. Greyish-black granites from Aswan granite quarries exhibit the lowest activity concentrations (^238^U = 20–74.4 Bq/kg; ^232^Th ≈ 60.6 Bq/kg; ^40^K ≈ 1200 Bq/kg), with absorbed dose rates of 66–118 nGy/h and Raeq values of 135–245 Bq/kg, all within international safety limits. Coarse pink granites from Aswan city quarries and Fila occurrence show moderate activity (^238^U = 12.4–74.4 Bq/kg; ^40^K = 1200–1900 Bq/kg), with anomalies such as sample 8A (^238^U = 496 Bq/kg; Raeq = 588.46 Bq/kg) reflecting localized enrichment in U-bearing phases (monazite). High-Dam granites display intermediate to elevated radioactivity (^232^Th ~ 84.8 Bq/kg; ^40^K ~ 1442 Bq/kg), with several samples exceeding hazard indices (Hex > 1), likely due to magmatic differentiation and structural concentration of Th-bearing minerals. Fine-grained granites are the most radiogenic; sample 12A yields ^238^U = 334.8 Bq/kg, ^232^Th = 214.12 Bq/kg, absorbed dose up to 334.52 nGy/h, Raeq = 733.86 Bq/kg, and Hex ≈ 2.9, making them unsuitable for indoor use and requiring strict monitoring.

**Fig. 24:**
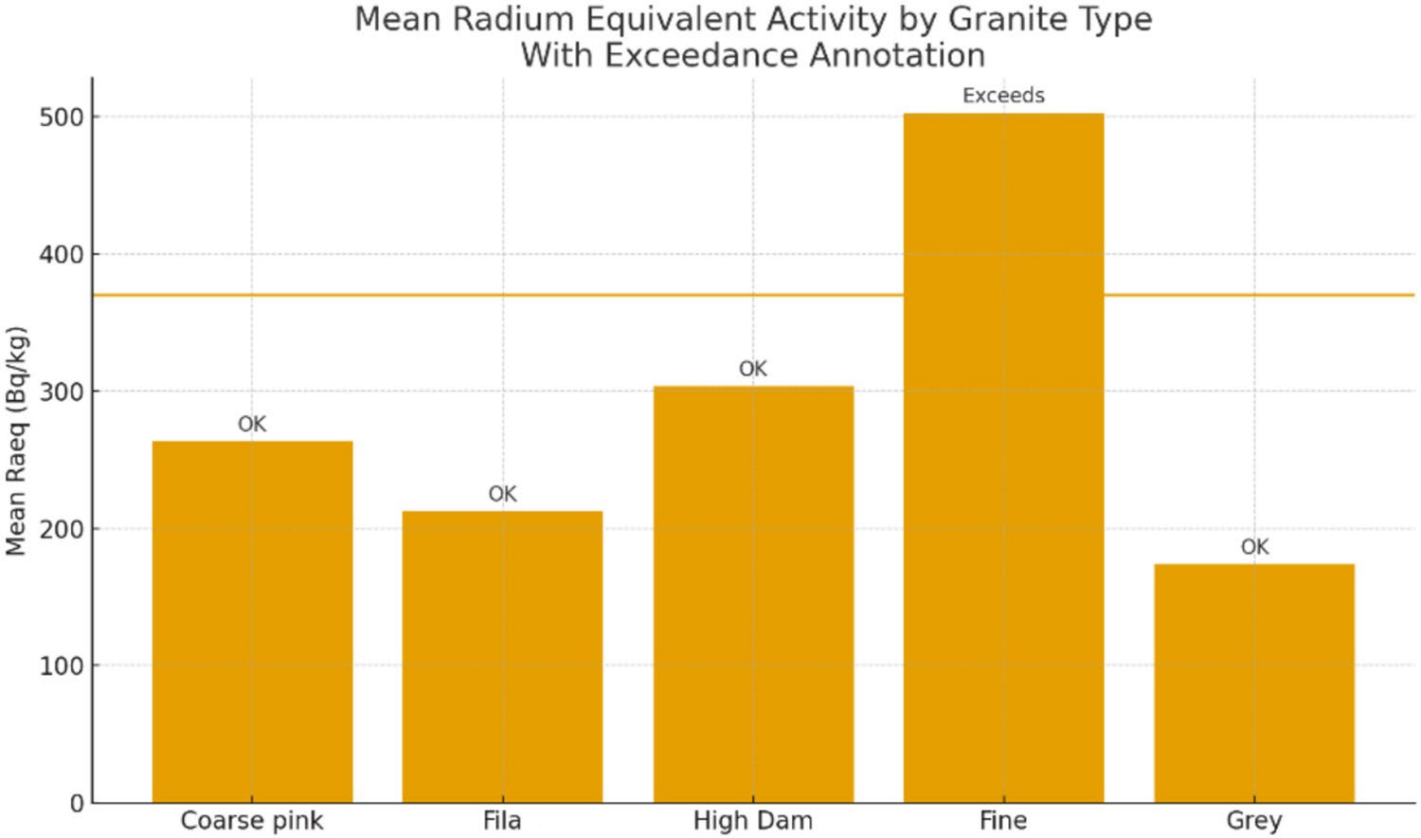
Mean Radium Equivalent Activity by granite type, with exceedance annotation.

Figure 24 illustrates mean radionuclide concentrations (^238^U, ^226^Ra, ^232^Th, ^40^K) against international thresholds. Fine-grained monzogranites exhibit the highest values; High-Dam granites show moderately elevated Th and K; coarse pink granites from Aswan city quarries and Fila occurrence remain largely within safe ranges except for localized U anomalies; grey granodioritic–tonalitic granites consistently fall below thresholds, representing the safest unit. Absorbed dose rates (65.8–334.52 nGy/h; mean 131.35 nGy/h) exceed global averages (~ 57 nGy/h), but annual effective doses (0.09–0.41 mSv/y; mean 0.16 mSv/y) remain below worldwide limits (0.48 mSv/y). Raeq, Hex, and Hin indices indicate localized areas requiring controlled quarrying and indoor-use restrictions.

Integration of hyperspectral and radiometric data highlights that greyish-black and Fila coarse pink granites are both mechanically reliable and radiologically safe for construction. Fine-grained and structurally deformed High-Dam granites require targeted radiological assessment, and quarrying in these units should be carefully managed to minimize health risks, geomorphic instability, and environmental degradation. These findings demonstrate the critical value of combining remote sensing and radiological analysis to guide sustainable quarrying and landscape management in the Aswan granite province.

Quarrying of Aswan granites is primarily controlled by texture, mineralogy, and structural fabrics. Coarse pink monzogranites and syenogranites—rich in K-feldspar, mechanically strong, and visually homogeneous—were favored for ancient monumental construction and remain highly valued today. In contrast, greyish-black tonalite–granodiorites, with higher mafic content and greater heterogeneity, yield blocks of variable quality and stability. Joint architecture exerts a key control on extraction: moderately spaced, planar N–S joints along the eastern Nile bank facilitate predictable block geometry and stable quarry faces. These structural advantages, combined with river proximity, explain the historical concentration of quarrying along the eastern margin, where ancient hand-cut pits coexist with modern mechanical operations.

Both ancient and modern quarrying illuminate the mechanical behavior and anthropogenic reshaping of Aswan granites. The Unfinished Obelisk offers unique insight into fracture propagation in coarse pink granite, where natural cracks, joint intersections, and nearby mafic enclaves halted extraction. This study provides the first detailed documentation by Professor El Bahariya of the obelisk’s geometry—42 m long, oriented N20° E, dipping ~ 30° SW, still attached to the host granite—while coarse mafic enclaves likely amplified stress concentration. Modern quarrying mirrors these lithological and structural controls: greyish-black granites are exploited where joint spacing allows large blocks, coarse pink granites remain the primary commercial resource, and fine-grained granites are selectively quarried for ornamental use, constrained by closer joint spacing and higher radioactivity.

Remote sensing reveals rapid expansion of quarrying, with slope removal, drainage alteration, and surface reflectance changes. Major sites include the Ibrahim Basha greyish-black quarry and extensive central Aswan pink-granite operations, including active Chinese-run sites. High-Dam granites, though aesthetically appealing, pose extraction challenges due to gneissose fabrics and anisotropic fracturing. Across all units, quarry-front attributes—grain size, enclave abundance, color uniformity, and fracture density—align with petrographic observations and govern block quality and economic value. Centuries of extraction have transformed the natural inselberg- and wadi-dominated landscape, producing pits, stepped faces, and modified drainage, with ancient quarry boundaries subdued yet still geomorphologically significant.

Overall, the discussion demonstrates that the Aswan granite landscape results from the integrated influence of magmatic evolution, structural control, geomorphological processes, human quarrying activity, and radiological behavior. Lithological and petrographic variability governs geomorphic resistance and landform development, while radiological signatures systematically reflect degrees of magmatic differentiation. Remote-sensing spectral responses closely match field and petrographic observations, enabling effective discrimination of granite types and monitoring of quarry expansion and landscape modification. Quarrying behavior consistently follows structural fabrics and textural properties, preferentially exploiting massive, coarse pink granites and reshaping the natural terrain over time. Together, these multidisciplinary observations confirm that the four granite types differ not only in origin and fabric, but also in geomorphic expression, radiological characteristics, and implications for safe use and sustainable resource management within the Aswan granite province.

## Conclusion and recommendations

This study provides the first integrated geological, petrographic, geomorphological, structural, remote-sensing, quarrying, and radiological assessment of the Aswan granite province. The results demonstrate that intrusive history, structural fabric, weathering, and human activities jointly control geomorphic evolution, fluvial patterns, quarrying potential, and radiological characteristics. This holistic framework underscores the importance of sustainable land-use and quarry-management strategies to safeguard the geological and cultural heritage of the Aswan region.

1. The Aswan granite province reflects multi-stage Neoproterozoic magmatism, Pan-African tectonism, and prolonged weathering and fluvial processes, producing four distinct granite suites: greyish-black tonalites–granodiorites, coarse pink monzogranites–syenogranites, High-Dam granites, and fine-grained granites.
2. Lithology, joint architecture, and structural fabrics (N–S joints, NE–SW shear zones) govern landscape evolution, Nile channel morphology, island formation, weathering patterns, and quarry block quality. Coarse pink granites dominate rugged terrain and islands; greyish-black granites are mechanically and radiologically safe; High-Dam granites show variable behavior; fine-grained granites are highly radiogenic, limiting indoor use.
3. Remote sensing, particularly PRISMA hyperspectral data, effectively discriminates granite types, maps shear zones, monitors quarrying impacts, and was validated through field and petrographic observations.
4. Radiological measurements reveal systematic variation in U–Th–K concentrations. While overall public exposure is low (< 0.48 mSv/y), fine-grained and deformed High-Dam granites require selective block-level screening, whereas coarse pink and greyish-black granites generally fall within safe limits.
5. Quarrying is controlled by lithology, texture, and structural features. Historical and modern extraction has reshaped landforms, altered drainage, and affected geomorphic stability.
6. Sustainable management of Aswan granites demands integrating geological, structural, geomorphological, radiological, and remote-sensing data to ensure safe construction, environmental protection, and heritage preservation.

The findings of this study underscore the need for responsible quarrying, informed land-use planning, and continuous environmental oversight in the Aswan granite province. Because geological, structural, geomorphological, and radiological factors are closely linked, effective management must integrate all these aspects. The following recommendations outline key actions for safe resource use, heritage protection, and long-term environmental sustainability.

1. Conduct routine radiological screening, with block-level testing for fine-grained and structurally deformed High-Dam granites and localized uranium-rich coarse pink zones.
2. Prioritize extraction from coarse pink and greyish-black granites; limit quarrying of fine-grained and highly sheared units.
3. Integrate hyperspectral/multispectral remote sensing for monitoring quarry expansion, slope stability, drainage changes, and environmental degradation.
4. Implement waste management and site rehabilitation to reduce erosion, dust, and geomorphic disturbance.
5. Incorporate lithological, structural, and radiological data into quarry licensing and land-use planning, especially near cultural and heritage sites.
6. Investigate radiogenic hotspots to identify U–Th–REE-bearing minerals and refine magmatic evolution models.
7. Develop geomorphology-based land-use guidelines focusing on slopes and terrains prone to instability.
8. Establish long-term multidisciplinary monitoring programs combining geological, geomorphological, radiological, and remote-sensing data to guide sustainable resource use and preserve Aswan’s geological and cultural heritage.

## Data Availability

The datasets used and/or analyzed during the current study are available from the corresponding author upon reasonable request.
